# Surface Design of Liquid Separation Membrane through Graft Polymerization: A State of the Art Review

**DOI:** 10.3390/membranes11110832

**Published:** 2021-10-28

**Authors:** Deepa Suresh, Pei Sean Goh, Ahmad Fauzi Ismail, Nidal Hilal

**Affiliations:** 1 Advanced Membrane Technology Research Centre, Faculty of Chemical and Energy Engineering, Universiti Teknologi Malaysia, Johor Bahru 81310, Johor, Malaysia; deepasuresh@graduate.utm.my (D.S.); afauzi@utm.my (A.F.I.); 2 NYUAD Water Research Center, New York University Abu Dhabi, Abu Dhabi P.O. Box 129188, United Arab Emirates

**Keywords:** membrane processes, chemical grafting, grafting-from, grafting techniques

## Abstract

Surface modification of membranes is an effective approach for imparting unique characteristics and additional functionalities to the membranes. Chemical grafting is a commonly used membrane modification technique due to its versatility in tailoring and optimizing the membrane surface with desired functionalities. Various types of polymers can be precisely grafted onto the membrane surface and the operating conditions of grafting can be tailored to further fine-tune the membrane surface properties. This review focuses on the recent strategies in improving the surface design of liquid separation membranes through grafting-from technique, also known as graft polymerization, to improve membrane performance in wastewater treatment and desalination applications. An overview on membrane technology processes such as pressure-driven and osmotically driven membrane processes are first briefly presented. Grafting-from surface chemical modification approaches including chemical initiated, plasma initiated and UV initiated approaches are discussed in terms of their features, advantages and limitations. The innovations in membrane surface modification techniques based on grafting-from techniques are comprehensively reviewed followed by some highlights on the current challenges in this field. It is concluded that grafting-from is a versatile and effective technique to introduce various functional groups to enhance the surface properties and separation performances of liquid separation membranes.

## 1. Introduction

All activities of mankind are reliant on water. Every day, hundreds of tons of wastewater are created from domestic usage, industrial, and agricultural activities. Water covers over three-quarters of the Earth’s surface, with seawater and brackish water accounting for 99% of the total and freshwater accounting for barely 1% [1,2,3]. Seemingly, freshwater resources are not replenished to satisfy the demands of a rapidly growing population, and this has also resulted in an unequal allocation of scarce freshwater resources across different industries [2]. Consequently, many people in the world, particularly those in underdeveloped nations, do not have access to safe drinking water. Agricultural activities are once again severely hampered, since farms lack sufficient water supplies for all-year irrigation and cattle production. These circumstances may be seen throughout the world, particularly in the Middle East, Africa, Asia, and Latin America. Statistics show that 2.1 billion people do not have access to clean drinking water at home, and over four billion people face acute water shortages for at least one month in a year [4]. One way of meeting freshwater demand is by reclaiming freshwater from the existing sources employing various wastewater treatment and desalination technologies.

Membrane technology emerged as a prevailing choice for commercial-scale desalination and wastewater treatment processes. Some widely used membrane processes for desalination and wastewater treatment include pressure-driven microfiltration (MF), ultrafiltration (UF), nanofiltration (NF) and reverse osmosis (RO). Recent years, osmotically-driven forward osmosis (FO) and thermally-driven membrane distillation (MD) also attract increasing attention from the desalination community. Membrane designs and modifications are at the forefront of research in this realm. Through proper design and introduction of functional groups, the separation efficiencies can be significantly enhanced. Membranes configured in the form of flat sheet or hollow fiber for water treatment and desalination have been improved through the development high-performance materials and advanced fabrication techniques. Membrane filtration involves interactions between the membrane surface and effluent molecules to be treated. The complex interaction takes place on the membrane surface also leads to a very unfavorable phenomenon known as fouling. Fouling causes a reduction in permeate flow and an increase in transmembrane pressure (TMP) [5]. Gradually, the membrane performance deteriorates and the cost of operation increases [6]. Membrane fouling can be mitigated by altering the membrane surface properties. For example, the increase in membrane surface hydrophilicity through the introduction of hydrophilic moieties during surface modification processes can render the membrane with greater fouling-resistance, while significantly improving the water flux [7].

Membrane surface modification adds new functionalities by changing the surface composition and structure without altering the desired macroscopic characteristics of the membranes. Through different types of surface modification, a broad range of properties such as wettability, roughness, dispersibility, and biological activities can be altered to meet the needs of the application [8,9]. Both physical and chemical approaches have been used for membrane surface modification. The physical method involves non-covalent interactions such as van der Waals force, hydrogen bonding, and electrostatic interaction between the modifying materials and the substrate surface. As physical approach can be performed in a single step using simple materials, this technique has gained wide recognition in commercial scale application [10]. Ontology doping, surface coating and layer by layer (LbL) assembly are a few examples of physical functionalization techniques [9]. Ontology doping of functional materials, particularly nanomaterials, is a physical method that involves a direct incorporation into the polymer matrices of membrane. Nanomaterial integration method usually relies on the amount of nanomaterial to enhance the desired properties and chemical functionalization magnifies the effect of modification by offering the molecular architecture [11].

Surface coating is a direct and inexpensive physical approach to alter the membrane surface. The surface structure is typically governed by hydrogen bonds and electrostatic interactions between the modifier and the membrane surface to keep the coating adhered to the membrane surface [12]. Surface coating via LbL assembly is a promising post-fabrication coating approach to create an ultrathin composite membrane layer with specific physicochemical properties [13]. This method is favorable to coat a wide range of polymeric or organic materials, and it allows a compact and highly dense layer with proper control over the layer composition and thickness [14]. Despite their feasibility, physical approaches are confronted by several limitations. The progressive leaching and exfoliation of the freshly deposited layer during operation is uncontrolled due to the weak non-covalent binding interactions [15]. A chemical approach is an appealing approach to overcome the limitation as it is enabled by covalent bond established through a number of chemical reactions [16]. The major limitation related to the incorporation of nanomaterials is the change in the integrity of the membrane structure. When the functional nanomaterials are introduced through ontology doping to the polyamide (PA) layer of thin film composite (TFC) membrane, the interfacial polymerization will be disrupted. This leads to the change in cross-linking degree and density of PA, and eventually change the structural characteristics of PA [17]. As a result, the increase in water flux has been normally achieved at the expense of the solute rejection ability. Furthermore, nanomaterial incorporation approach relies on a high loading of nanomaterial to amplify the effect of alteration [18]. For example, the water contact angle of the modified membrane is reduced proportionally to the amount of nanofiller incorporated. 

Membrane surface modification through chemical grafting strategy is gaining popularity owing to its versatility in customizing desirable surface characteristics with various monomers. Chemical grafting is a versatile approach to change the surface of a membrane by creating a “tailored” membrane surface with desired functions. A single monomer or a mixture of monomers can be used for grafting. The desired functional molecules or chains can be precisely grafted onto the desired location of the membrane. Ultraviolet (UV) irradiation and plasma treatment are commonly used to activate the membrane surface [3,19,20]. Grafting of hydrophilic materials helps to improve separation performances in terms of rejection and water flux as well as the anti-fouling and chlorine resistance properties [3,21]. Typically, differences in the type of reactant, the number of functional groups, or the modification technique can generate layers of varied topologies in chemical grafting [22].

Many advances have been made in the modification of membranes through chemical grafting. In response to the progresses and development made in this field, a number of reviews related to chemical grafting of polymeric membranes have been published. Uyama and group [23] described various grafting procedures and grafted surface characterizations in general. Li and colleagues reviewed the “grafting-from” polymerization of zwitterionic monomers on the surfaces of polyvinylidene fluoride (PVDF) and polyether sulfone/polysulfone (PES/PSF) membranes [24]. The development of anti-protein fouling materials based on hydrophilic zwitterions was highlighted. Lee and group presented the pros and cons of different grafting methods employed for the surface modification of membranes used in membrane bioreactor [25]. Pinem et al. provided a mini review on chemical grafting approaches for liquid separation membrane where different types of grafting-from approaches have been briefly discussed [26]. Despite the efforts made in this topic, the elaborations on the modification strategies and the corresponding membrane performances upon grafting modification have not been reviewed in the previously published work. 

Acknowledging the current gaps, this article emphasizes the surface design of liquid separation membrane through grafting-from, a chemical grafting technique, to enhance membrane performances for wastewater treatment and desalination. Grafting-from technique is known as one of the most versatile approaches for membrane surface modifications. The primary focus of this review is to elaborate the roles of grafting-from surface modification technique in minimizing membrane’s inherent issues thereby enhancing membrane performance. In the following sections, membrane-based separation processes for liquid separation including pressure-driven and osmotically-driven processes and the general membrane surface modification approaches are briefly discussed. Membrane modification based on chemical grafting techniques are discussed in detail in terms of their classifications, features and advantages. The grafting-from chemical modification techniques is discussed with emphasis put on their advancements and limitations in rendering desired membrane properties and performance. The perspectives provide some references to researchers to further investigate grafting-from technique for enhanced water treatment processes and promote the development of this approach for membrane surface modification.

## 2. Brief Overview on Membrane Technology

Membrane processes rely on a semipermeable polymeric or inorganic membrane with a specific structure to separate a mixture. Pressure driven membrane processes i.e., microfiltration (MF), ultrafiltration (UF), nanofiltration (NF) and reverse osmosis (RO) are prevailing processes for liquid separation, particularly for wastewater treatment and desalination. MF membranes can separate large suspended particles including colloids, particulates, lipids, and bacteria [27]. Although MF is incapable of eliminating dissolved particles smaller than 1 mm and it is not an impenetrable barrier to viruses, it aids in the management of harmful microorganisms in water when used in disinfection. UF is widely used to eliminate pathogenic microorganisms, macromolecules, and suspended debris [28,29]. Most MF and UF membranes are asymmetric with a thin skin layer and a polymeric support layer for separation. The polymeric support layer is generally composed of semi-hydrophobic polymers such as PSF, PVDF, and PES [30]. NF operates at lower pressure than RO and it can reject organic compounds and almost all multivalent ions while moderately reject monovalent ions. NF finds wide applications in brackish water desalination and dye removal.

Reverse osmosis can effectively eliminate all monovalent ions while permitting water molecules to flow through. RO membranes feature pore structure that is significantly compact than that of other conventional pressure-driven membranes, allowing them to remove almost all particles, bacteria, and organics. An optimal RO membrane can reject more than 99% of salt and 99.9% of organics hence has been reliably used for seawater desalination. However, due to the high-pressure requirement, RO is more costly than other membrane systems and more vulnerable to fouling. Therefore, a high degree of pretreatment is generally required to combat RO membrane fouling issue [31]. Forward osmosis (FO) is driven by osmotic pressure difference created by the solute concentrate difference between the feed solution and draw solution [32,33]. FO is capable of rejecting a wide range of inorganic and organic constituents. Compared to RO, FO membrane normally suffers from a less severe irreversible fouling. RO and FO membranes are typically configured in the form of thin film composite in which a PA selective layer is interfacial polymerized on a microporous substrate which provides mechanical support to the thin selective layer [34,35]. For FO membrane, the substrate should have high hydrophilicity, high porosity and low tortuosity to minimize internal concentration polarization (ICP) in FO membrane.

Membrane distillation (MD) is a thermally driven separation technique that separates non-volatile chemicals using a hydrophobic microporous membrane [36] with pore sizes ranging from 0.1 to 1 m and porosities ranging from 40% to 90% [37,38]. The hydrophobicity of the membrane hinders liquid mass transfer, resulting in the formation of a gas-liquid interface [39]. MD membranes can theoretically reject 100% of non-volatile solutes to yield water with high purity [40] therefore it has a variety of applications, including desalination and nutrient recovery [41]. MD membranes should have high thermal stability, chemically resistance and high liquid entry pressure (LEP) [38,42]. The fouling and scaling of MD are highly dependent on the parameters of the feed water and foulants, as well as the membrane qualities and operational circumstances such as feed temperature and flow velocity [43]. Although fouling and scaling are less bothersome in MD compared to that of. in pressure-driven separation processes, the phenomenon must be carefully addressed to avoid unnecessary additional treatment and membrane replacement costs [44]. 

## 3. Surface Chemical Grafting on Polymeric Substrates 

### 3.1. Classification of Chemical Grafting

**Figure 1:**
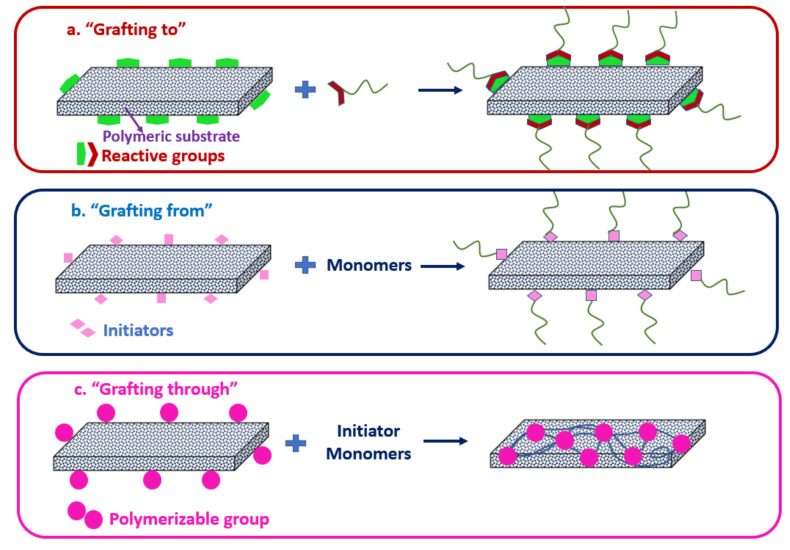
Strategies of polymer grafting: (**a**) grafting-to; (**b**) grafting-from; (**c**) grafting-through.

Tethering hydrophilic polymer chains through chemical grafting is a promising method to enhance the surface hydrophilicity and anti-fouling resistance of the membrane. Grafting-to, grafting-from and grafting-through are prevalent chemical grafting techniques used for surface modifications. As illustrated in Figure 1a, preformed polymer chains are attached to the substrate surface by covalent bonding in the grafting-to or polymer grafting method, without involving any polymerization reaction. In most cases, a condensation reaction occurs between the polymer chains’ terminal functional groups and compatible reactive sites that are produced at arbitrary on the substrate surface. Due to the steric effects of the bulky grafted polymeric chains, grafting-to method usually results in polymer brushes with low surface density [45]. Grafting-to is considered as a straightforward grafting technique as the polymers grafting can be performed in single step. Direct grafting and bridging agent meditated grafting are examples of grafting-to approach. Liu et al. reported improved membrane permeability and anti-fouling properties through the direct surface grafting of lysine (Lys), a short chain amino acid with immense hydrophilicity [46]. Besides, Yi et al. showed that the meditated grafting of nitrogen-doped graphene oxide quantum dots onto the PA TFC RO membrane enhanced water flux and chlorine resistance of the modified membrane [47].

In the grafting-from or graft polymerization technique, monomers gradually extend from the grafting sites of the substrate backbone to form side chains of customizable lengths, as illustrated in Figure 1b. Monomers are introduced to the surface initiation sites during the grafting process to facilitate the formation of a high-density polymer sheet [48]. Through grafting-to approach, the formation of homopolymers can enhance the polydispersity of the polymer sheet [45]. Grafting-from technique is useful in managing the thickness of the grafting layer as the monomer concentration can be increased over time. However, it is arduous to manage the final chain length of the responsive material using this approach [49]. Surface initiation sites required for graft polymerization can be produced by using a chemical initiator, UV/gamma irradiation, or plasma [50]. Transfer radical polymerization (ATRP) and plasma-initiated graft polymerization are the two most commonly used methods for substrate surface activation. The radicals formed by initiators strike the polymer backbones and initiate the reaction during polymerization. Polymer propagation forms a film that cover-up the substrate surface. The thickness and coverage of the grafted polymer can be increased by enhancing the grafted period, monomer concentration, and initiator concentration [51].

Grafting-through, as illustrated in Figure 1c, is another method for creating well-specified side chains. A lower molecular weight monomer is often copolymerized with free radicals with an acrylate functionalized macromonomer. The ratio of monomer to macromonomer molar concentrations, along with their copolymerization activity, influence the number of the chains grafted. The number of chains grafted is determined by the ratio of monomer to macromonomer molar concentrations as well as their copolymerization activity [52]. As the reaction progresses, the ratio of monomer to macromonomer change, resulting in random branch placement and the generation of graft copolymers with varying branch counts. Based on the reactivity ratio of the macromolecular terminal functional group to the monomer, this approach allows heterogeneous or homogeneous branch addition [53]. The physical characteristics of the grafted copolymer are affected by the differences in graft distribution. Any known polymerization procedure can be used for the grafting-from approach. Among the three grafting approaches, grafting-from technique provides the greatest flexibility to tailor the surface properties of a liquid separation membrane [54]. In the following sub-section, the common techniques used for the accomplishment of chemical grafting-from for polymeric substrate, particularly for polymeric membrane surface, are discussed.

### 3.2. Chemical-Induced Graft Polymerization

**Figure 2:**
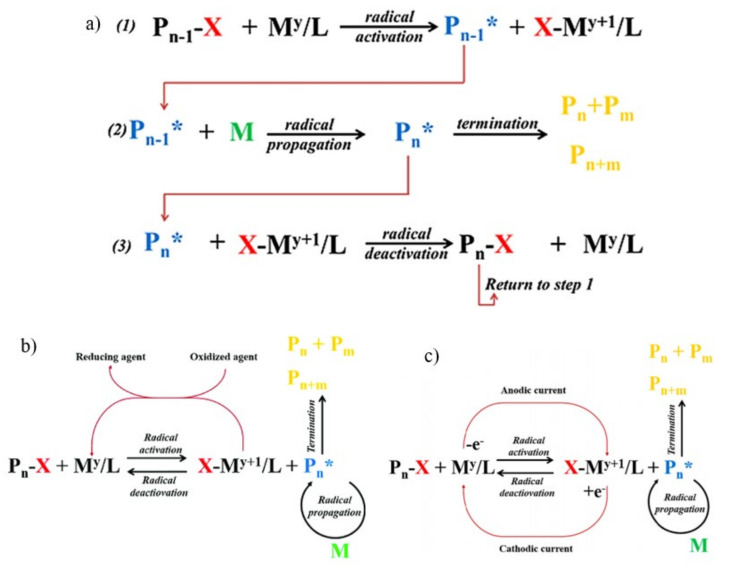
General mechanism of (**a**) traditional atom transfer radical polymerization in three simple steps; (**b**) AGET-ATRP; (**c**) SI-eATRP [56].

Chemical-induced graft polymerization involves the development of a radical on the membrane surface due to its interaction with a chemical-based surface activator. ATRP is a living radical polymerization method that can polymerize ample range of monomers to produce polymers with precisely regulated molecular weight, molecular architecture, and polymer structure [55]. The atom transfer step is critical in the process that results in uniform polymer chain development. ATRP requires the activation of the initial alkyl halide adduct formed with an unsaturated molecule (monomer) and the subsequent reactivity of the intermittently generated radical with more monomer units (propagation). An alkyl (pseudo)halide, which might be a low or high molar mass chemical, or even a component of an insoluble substance, is generally used as the initiator. These initiators are generally bind to the surface of modified particles, flat wafers, or fibers. Figure 2a depicts the overall mechanism of this controlled radical polymerization, in which radicals and dormant alkyl halides establish a rapid dynamic equilibrium [56]. ATRP activates a “dormant” macro alkyl halide (Pn-1–X) (step 1) via a catalytic (My/L, M usually is a transition metal; commonly Cu, and L is a ligand) process, resulting in a complex with a higher oxidation state (X–My + 1/L) and a (macro-radical) “living” polymer (Pn*). According to step 2 (Figure 2a), the “living” polymer propagates and can undergo chain transfer or bimolecular termination, resulting in the polymer becoming “dead” (inactive). The “living” polymer is deactivated back to “dormant” polymer (macro alkyl halides) in step 3 after a single or multiple monomer addition, preventing bimolecular terminations [57]. A common ATRP approach for PA TFC membrane modification is immobilizing ATRP initiators on PA structures followed by the grafting of functional polymers. Ginic-Markovic et al. applied ATRP grafting to introduce polysulfobetaine onto the surface of commercial RO membrane to enhance the hydrophilicity and smoothness of the membrane surface for anti-fouling purpose [58]. Yang et al. demonstrated the efficiency of SI-ATRP grafted amphiphilic TFC RO membrane in repelling sodium alginate and bacteria foulants [59]. However, ATRP is a complicated reaction in many ways because it involves one or more (co)monomers. It also involves the metal complex transformation in two or more oxidation states which may contain numerous counterions and ligands. The efficiency of ATRP is highly dependent on the chemicals and operating conditions [60]. The components in the reaction media can potentially affect the interactions between the reagents and alter the ATRP equilibrium [57]. The multi-step modifications are also time-consuming. ATRP initiators activate on the backbone of an acyl chloride or amino groups might deplete the inter-chain hydrogen link between PA skeletons, resulting in a decrease in selectivity [61]. In addition, the ATRP system is not reusable, the reaction must take place in an inert environment, and the toxicity of metal catalysts makes the process less ecologically beneficial [62]. Therefore, other grafting approaches have been made to address the limitations of conventional ATRP process.

Activators regenerated by electron transfer atom radical polymerization (ARGET-ATRP) is known to be a greener process as it employs considerably lower catalyst concentrations in a system that is appropriate for commercial scale up and the grafting processes tends to generate pure α-functional products [56]. The advantage of ARGET-ATRP is that copper catalyst-induced side reactions, notably for acrylates [63], are significantly decreased to allow for a much greater conversion of the ATRP process and the production of copolymers with a much higher molecular weight while sustaining chain end functionality [64]. This has been verified by the successful chain extension of macromolecules produced utilizing ARGET-ATRP [65]. In an ARGET-ATRP method, catalyst selection is critical to achieve controlled polymerization, and an accelerated approach for catalyst optimization has been investigated [65]. For copper (CuI) regeneration, ARGET-ATRP uses non-radical producing reducing agents. A good reducing agent (such as hydrazine, phenols, sugars, or ascorbic acid) can exclusively react with CuII and not with other reagents in the reaction mixture. ARGET-ATRP can be initiated with the more easily manipulable oxidative stable catalyst species (X-My + 1/L). As X-My + 1/L may be reduced in situ, a catalyst in the active state (My/L) can be obtained as in Figure 2b [56]. 

Surface-initiated electrochemically mediated atom transfers radical polymerization (SI-eATRP) initiates/restricts the controlled/living ATRP chain propagation process by electrochemically producing the activator (lower-oxidation-state metal complex) from deactivator (higher-oxidation-state metal complex). The SI-eATRP has been touted as an effective approach for the synthesis of polymers with complex structures such as stars, brushes, and block copolymers due to the degree of control over the reaction [66]. A cathodic current is used to produce My/L catalytic complex in the SI-eATRP process mechanism as shown in Figure 2c. An optional anodic current is also utilized to stop the polymerization process by reverting the active catalyst state to the X-My + 1/L complex. After reduction, the My/L complex is disseminated in the polymerization medium by rapid stirring [67]. Polymerization rates and concentrations of active catalytic species may be manipulated by adjusting current, voltage, and total charge passed. Wu et al. demonstrated mediating the reaction state of SI-eATRP grafted polymer brushes significantly regulate polymerization on the polymer membrane surface [68].

Reversible addition-fragmentation chain transfer (RAFT) is a simple reaction that may be performed using a variety of monomers and solvents under mild reaction conditions. Song and colleague used surface-initiated RAFT polymerization to build 3-D nanolayers on a porous anodic aluminum oxide-silica composite membrane [69]. Quaternized poly (3 methacrylamidomethyl)-pyridine chains were used to make polyelectrolyte brushes that formed consistent hydrophilic pores. Kochameshki and group used xanthate RAFT polymerization to insert poly (diallyldimethylammonium chloride) on graphene oxide membranes, making the top surface highly hydrophilic, and evaluated the impact of alteration on perm selectivity [20]. 

Ozonation-initiated grafting is a less favorable option due to the presence of concentrated O_3_ that can harm human and animal health as well as vegetation at lower altitudes [70]. Ozone or oxidizing agents such as benzoyl peroxide (BPO) and benzophenonyl bromoisobutyrate (BPBB) have been effectively used as a precursor to modify polypropylene (PP) membranes through the grafting of hydroxyethyl methacrylate (HEMA) [71,72], N,Ndimethylaminoethyl methacrylate (DMAEMA) [73], and acrylic acid (AA) [74]. The chemically grafted PP membrane showed enhanced hydrophilicity. 

### 3.3. Plasma-Induced Graft Polymerization

Plasma is a state of matter made up of a mix of ions, electrons, excited species, UV irradiation in vacuum, and free radicals [3,75]. Two major processes, i.e., breakdown of a polymer support and formation of a new modified layer on the membrane surface occurs when plasma interacts with a polymer membrane. The balance between these membrane processes is determined by the type of plasma gas used and applied process parameters. Plasma induced membrane modification is a quick method that produces uniform grafts on the membrane’s surface by inducing four basic effects: cleaning, cross-linking, ablation, and chemical alteration [75,76]. The change in surface energy caused by plasma induced grafting has a remarkable effect on membrane fouling [77].

**Figure 3:**
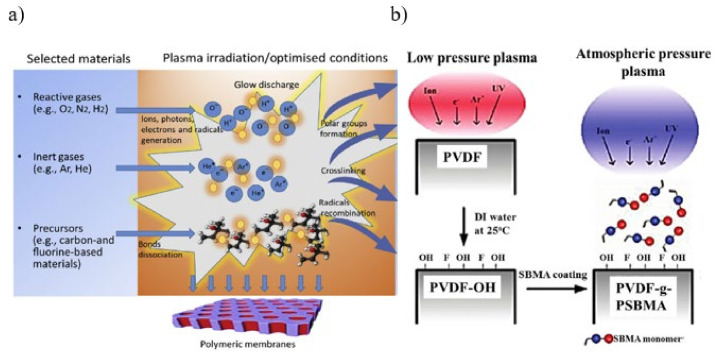
Schematic illustration of (**a**) plasma modification of polymeric membrane [84]; (**b**) Zwitterionic polyvinylidene fluoride (PVDF)-g-PSBMA membranes prepared by atmospheric plasma-induced surface copolymerization [51].

Plasma treatment, in general, can be used directly or indirectly to modify polymeric surfaces by forming functional groups. Direct treatment consists of the use of reactive plasma gases such as NH_3_, O_2_ are known to create desired functionalities like amines, COOH, and free radicals [78]. The parameters, such as treatment duration, pressure, power, and processing gas, as well as the nature of the irradiated surface, will influence the subsequent alteration for both direct and indirect treatment types [79]. The formation of plasma can be carried out using water, noble and non-polymerizing gases such as helium (He), argon (Ar), neon (Ne), tetrafluoromethane (CF_4_), oxygen (O_2_), hydrogen (H_2_), carbon dioxide (CO_2_), nitrogen(N_2_), water and air as shown in Figure 3a. The radicals formed in plasma membrane primarily attack C-C, C-H, and C-S bonds in their plasmatic state [80]. These radicals, on the other hand, do not damage aromatic C-C and C-H bonds [81]. The radicals interact with particular functional groups originally present on the membrane surface during the treatment phase, enhancing the grafting process [82]. The active species produced in the plasma zone can enhance biological compatibility, adhesion, and wettability characteristics by activating the membrane’s top molecular layer without altering the bulk of the polymer [77]. Plasma induced grafting can be considered as an environmentally friendly approach because it does not entail the use of any wet or harmful chemicals [83]. Nonetheless, to prevent the degradation of surface functionality, the grafting process must be carefully controlled, particularly in terms of the orientation of both polar and chain groups. If the plasma treatment is not functioning accordingly, a phenomenon acknowledged as “hydrophobic recovery” might occur, causing the grafting process to reverse. Excessive exposure to plasma can damage the membrane polymer [84].

Different power sources have been investigated to generate plasma, including radiofrequency, microwave, photons, direct current (DC) of high voltage, and low-pressure DC glow discharge [85]. Power and gas are essential to setup the grafting process and the operating condition should be kept at a suitable level while utilizing plasma-induced graft polymerization. There will be more active sites on the membrane surface as the temperature rises, and monomer diffusion will improve as the grafting degree rises. Due to the higher viscosity, a high-concentration monomer may limit the degree of grafting by restricting monomer diffusion to the membrane surface [85] or preventing plasma treatment from reaching the PP membrane surface, resulting in little or inefficient active site formation [86]. Different chemical functional groups can be brought to the membrane surface depending on the gas employed. For instance, N_2_ plasma occur in the presence of amide, amine, imine and nitrilite chemical groups while water plasma take place in the presence of hydroxyl, carboxyl and carbonyl chemical groups [80]. The combinations of different plasma and polymer result in various membrane surface chemistries. Changing the process variables allows plasma induced grafting to generate a range of hydrophilic membranes. Additionally, the two primary processes in plasma-induced grafting of poly(zwitterion) chains on the membrane surface are the activation of the polymer support by plasma (production of radicals) and the deposition of a new zwitterionic layer on the membrane surface via polymerization [87]. The poly(zwitterionic) layer thickness may be adjusted to the angstrom level [88]. The dynamic plasma flow also produces more peroxides on the membrane and even stimulates the underlying membrane layer, as well as having a longer glow distance than the static plasma [89]. Figure 3b shows the schematic of the formation of zwitterionic polyvinylidene fluoride-graft-poly sulfobetaine methacrylate (PVDF-g-PSBMA) membrane using atmospheric plasma-induced surface copolymerization.

Numerous techniques have been used to activate the surface using atmospheric pressure plasma, namely arc discharge, corona discharge, dielectric barrier discharge and its variation piezoelectric direct discharge. The use of arcs and plasma torch for polymer surface activation is not recommended since the high electron temperature might cause etching and damage to the surface. As a result, for polymeric substrates, dielectric barrier discharge and impinging jet have been preferred. Kim et al. devised a two-step procedure to propose polyacrylic acid (PAA) onto the surface of PA TFC RO membrane [90]. The PA layer was initially activated with an atmospheric pressure helium plasma treatment to produce high-density surface-active sites. Following that, aqueous phase free-radical polymerization of AA was promptly performed after surface activation to bind PAA chains to the surface.

### 3.4. Irradiation-Induced Graft Polymerization

Irradiation induced grafting has been a topic of investigation for researchers especially for membrane modification due to the grafting features such as quick processing, homogeneous reaction system, direct initiation without additive, temperature-independent process and direct cross-linking [91]. UV, electromagnetic photons (gamma rays, X-rays), and charged particles (electron beam and swift heavy ions) are examples of sources of radiation [92]. Both radioactive isotopes such as Cobalt-60 (Co-60) and Cesium-137 (Cs-137) may produce gamma rays. Co-60 gamma radiation offers benefits such as greater energy emission (1.25 MeV), cheap cost, and simplicity of generation. However, the highly energetic gamma rays may destroy chemical bonds, degrade membrane strength, and produce radioactive waste, thus limiting their use.

UV irradiation was the most often employed induction for grafting polymers or additives onto membrane surfaces. UV-assisted grafting provides particular benefits in terms of its simplicity, affordability, and application range. This straightforward approach enhances membrane surface wettability while narrowing the membrane pore size distribution. A photo-initiator adsorbed onto the polymer surface is selectively UV excited, resulting in heterogeneous hydrogen abstraction and subsequent activation of polymer surface modification processes by polymer processes [93]. Surface modification is performed in this fashion to graft hydrophilic monomers onto hydrophobic membranes such as PES and PSF using a relatively simple, low-cost graft polymerization process [94]. UV grafting approach can be improved by depositing polyelectrolyte monomers onto the membrane surface during the grafting process, resulting in a negative charge on the membrane surface. This technique has been to minimize natural organic matter (NOM) fouling by controlling electrostatic interactions at the membrane surface as well as hydrophobic/hydrophilic interactions [95]. 

**Figure 4:**
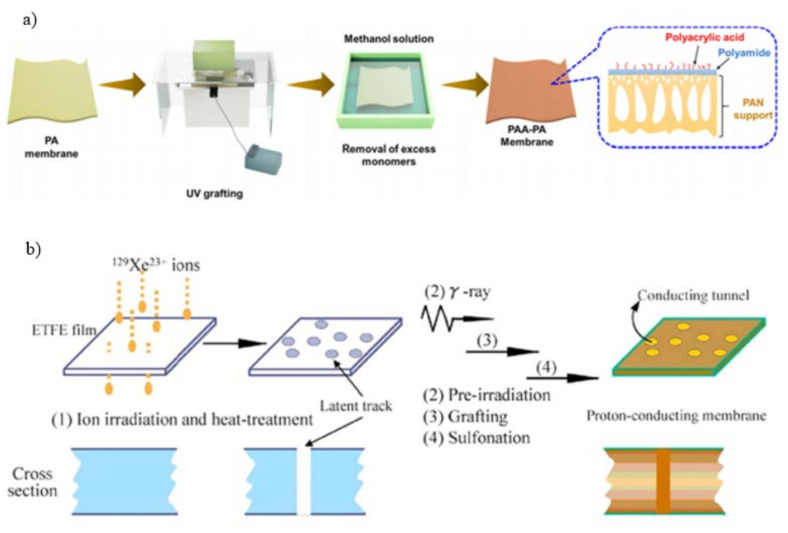
Schematic representation of (**a**) Preparing UV-grafted composite membrane [98]; (**b**) Irradiation-initiated graft polymerization on membranes [108].

UV-initiated graft polymerization has been used to modify membrane surfaces with several monomers such as 2-acrylamidoglycolic acid (AAG), AA, HEMA, AAM (Acrylamide), and N-vinyl-2-pyrrolidinone (NVP). These monomers make the membrane surface more hydrophilic, thus less prone to fouling [96]. The simplest unsaturated carboxylic acid, AA, is an organic molecule with a vinyl group directly connected to a carboxylic acid terminal [97]. The process for preparing PAA grafted PA layer through UV-initiated graft polymerization is shown in Figure 4a [98]. Parameters such as the concentration of AA, the irradiation period, and the irradiation distance can be varied accordingly. The presence of chlorine-susceptible sites of amide linkages and end amine groups in aromatic PA chains is reduced when this monomer is bind to the membrane surface, enhancing the chlorine stability of this layer [99]. In an investigation utilizing UV irradiation for the grafting of 374 µg/cm^2^ ((3-methacryloylamino) propyl) dimethyl(3-sulfopropyl) ammonium hydroxide (MPDSAH) zwitterion ion using benzophenone as the initiator, it was shown that the modified membrane experience 30% decrease in the total flux loss and a 44% decrease in the irreversible flux loss [100]. Surface modification of RO PA commercial membranes has been performed using AA in combination with other monomers such as N-isopropyl-acrylamide (NIPAAm). Upon NIPAAm and AA graft polymerization, the surface of these membranes became more hydrophilic and negatively charged [101].

It has been reported that UV irradiation was used to graft hydrophilic and fouling-resistant poly 2-[(methacryloyloxy)ethyl] dimethyl-(3-sulfopropyl) ammonium hydroxide (PSPE) [102] and PEG [103] to the surface of PA TFC. To improve the anti-fouling capabilities of the PA TFC RO membrane, Asadollahi et al. used UV-initiated grafting of acrylamide mixed with TiO_2_ nanoparticles [102]. Due to the overwhelming cross-linking action over chain scission, selective layer densification was seen after a short irradiation duration of <90 s. Due to the inclusion of hydrophilic TiO_2_, the membrane’s salt rejection ability enhanced without affecting the water flow. The increase in membrane selectivity is due to UV’s ability to induce cross-linking in the polymer matrix [103]. Therefore, UV irradiation has been frequently utilized for membrane production or modification.

Research on the application of gamma ray-induced grafting is also ongoing. The monomer content and gamma ray dosage rate are the major factors that determine the membrane characteristics in this grafting. In common, increasing the monomer concentration and dosage rate enhance the grafting until the optimal states are attained. The processes of radiation-induced grafting are known as simultaneous irradiation and pre-irradiation [92,104]. The monomer is directly grafted to the polymer membrane in a single reaction step under concurrent irradiation while displaying the membrane backbone to free radicals under vacuum or inert circumstances, followed by disclosure to the liquid or vapor states of monomers occur under pre-irradiation [105,106]. Figure 4b depicts a schematic representation of the radiation-induced grafting process (Xe-irradiated), which includes four key processes: ion irradiation, pre-irradiation, grafting, and sulfonation. Electron-beam induced grafting has also been carried out on the PES membranes to graft several functional molecules on its surface [107]. The water contact angles of the modified membranes were in the range of 24–54° for different types of functional group in the following order: PO_3_H > -NH_2_/-OH > -SO_3_H > -CO_2_H. This provides a guide to identify the suitable functional group in increasing membrane’s hydrophilicity.

The utilization of a mixture of several grafting approach has gotten a lot of interest in recent years. For instance, UV/ozone induced grafting can be utilized to increase membrane hydrophilicity. This method was tested on pulsed-electric fields (PEF) UF membranes using several graft materials such as polyethylene glycol (PEG), polyvinyl alcohol (PVA), and chitosan [109]. Among the grafted membranes, PEF-PEG demonstrated the best antifouling capabilities based on surface roughness and protein adsorption data. In another work, Li et al. (2017) used heat started grafting followed by ozone modification to graft sodium styrene sulfonate (SSS) monomer onto PVDF [62]. The water contact angle was considerably changed by manipulating the PVDF/SSS ratio, and with an increase in SSS, the surface hydrophilicity was increased. The results were consistent with the water absorption test, which showed an increasing trend from 5% to 25%. The capacity was increased by nearly 5 times above what it was before. Table 1 summarizes the grafting polymerization of several types of polymeric membrane via the above-mentioned grafting methods, and the corresponding characteristics of the modified surface. Chemical and plasma induced graft polymerization has been utilized to enhance the hydrophilicity of PP and PES membrane while irradiated induced graft polymerization has been utilized to improve the hydrophilicity of PP, PES, Polyarylsulfone (PAS) and PVDF membranes. Due to the presence of the monomer graft, polymeric membranes modified through chemical-induced graft polymerization are endowed with the desired physio-chemical properties.

In a nutshell, graft polymerization is a potential approach to effectively introduce functional groups that can enhance membrane hydrophilicity, hence the water flow and the fouling resistance of the membranes for wastewater treatment. In addition, membrane alteration also boosts the biocompatibility of membrane processes, allowing them to be used in more biomedical applications. In common, the grafting degree determines the properties of modified membrane. For instance, the higher degree of graft polymerization provides a powerful driving force for the hydrophilic monomers to migrate and reorient, lowering the interfacial free energy [117]. Subsequently, a high grafting degree combined with a wide covering area reduces the water contact angle. Xu et al. reported that the water contact angle of cellulose membrane reduced from 28° to 13° when the grafting degree was increased [118]. Pourziad et al. also observed the decrease in water contact angle of PVDF UF membrane from 96° to 58° when the amount of grafted NIPAAm decreased from 0.11 mol/m^2^ to 0.0149 mol/m^2^ [119]. It has also been generally observed that, when the grafting degree was low, the water flux on the modified membrane remained nearly unchanged. On the other hand, as the grafting degree increased, pore-covering became a major issue, causing the water flux to decrease dramatically. Accurate graft layer geometry and well-controlled graft architecture are critical to optimize the properties of the grafted membrane.

## 4. Liquid Separation Membrane with Chemically Grafted Architecture: Performance Evaluation

Polymer grafting can be feasibly performed on asymmetric integrally skin membrane and PA TFC membrane used for liquid separation. However, the surface activation, grafting procedure, and grafting molecules can be varied depending on the surface reactivity of the membranes [120]. In the following sections, the roles of graft polymerization in altering membrane surface properties and separation performances are discussed in detail based on two main configurations of liquid separation membranes, i.e., asymmetric integrally skin membrane and thin film composite membranes.

### 4.1. Asymmetric Integrally Skin Membrane

An asymmetric integrally skin membrane typically made up of a thin skin layer (0.1–10 µm) supported by a much thicker macroporous sublayer with anisotropic structure. The commonly used polymeric materials used for the preparation of asymmetric integrally skinned liquid separation membranes such as PVDF, PSF and PES are reactive towards most types of graft polymerization techniques. 

PVDF UF membrane was modified by vinylimidazole (VIM) and subsequently grafted with PAA through SI-ATRP polymerization [121]. The activated halide groups of the copolymer component of PVDF UF membrane reacted with VIM to produce PVDF-VIM membranes with polymerizable moieties poly (methyl methacrylate-*co*-4-chloromethyl styrene-vinylimidazole (PMMA-co-PCMSt-VIM). Increased grafting time up to 4 h altered the surface morphology of the modified membranes to form a smooth and featureless surface. The rise in grafting density with treatment time resulted in more grafted PAA. To produce hydrophilic feature, the surface characteristic of a base membrane as well as the resulting modified membrane is highly important. When the membrane was grafted with PAA chains, a rough surface and optimized grafting time are necessary to allow better anchoring and induce strong anti-fouling properties to the membrane for oil-in-water separation and protein filtering. With grafting duration of 2–4 h, excellent antifouling properties were attained as observed from the flux recovery. 

**Figure 5:**
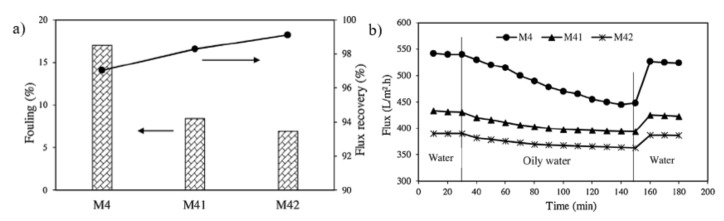
(**a**) Fouling ratio and flux recovery ratio of modified membranes (M4, M41, M42) where M4 = PVDF-g-PNIPAAm membrane (Polymerization time: 20 h), M41 = PVDF-g-PNIPAAm-b-PPEGMA membrane (Polymerization time: 8 h), M42 = PVDF-g-PNIPAAm-b-PPEGMA membrane (Polymerization time: 16 h); (**b**) Time-dependent flux for modified membranes (M4, M41, M42) during filtration of synthetic oily water at 700 kPa and 0.03 m/s 40 °C [119].

The graft polymerization of poly(N-isopropylacrylamide) (PNIPAAm) and PEGMA have also been performed via SI-ATRP grafting on commercial PVDF UF membranes for oily wastewater treatment [119]. These co-polymers rendered the membranes with excellent fouling resistance and self-cleaning properties. The bottom block of NIPAAm was grafted to give the membrane temperature sensitivity, while the top block of PPEGMA was utilized to enhance hydrophilicity. Due to the increased hydrophilicity of PVDF-g PNIPAAm-b-PPEGMA membranes, the fouling ratio of PNIPAAm-b-PPEGMA modified membranes with polymerization time of 8 and 16 h was lower than that of PVDF-g-PNIPAAm membranes with polymerization time 20 h, as shown in Figure 5a. This improvement was due to the presence of hydrophilic PPEGMA brushes on the membrane surface which has reduced the adhesion of oil molecules to the membrane surface [122]. When compared to commercial PVDF membranes, PVDF-g-PNIPAAm-b-PPEGMA membranes exhibited a slightly greater FRR. As a result of grafting PEGMA chains, FRR has not altered considerably. However, as shown in Figure 5b, despite the increase in surface hydrophilicity, the permeation flux of PVDF-g PNIPAAm-block-PPEGMA modified membranes was lower than that of pristine membranes due to a more dominant pore blockage effect. The prolonged PPEGMA polymerization duration lowers the permeation flux up to 365 Lm^−2^ h^−1^. The number of grafted PEGMA chains increased when the PEGMA polymerization time was increased, resulting in more pore blockage and lower flux values. Membranes grafted with PNIPAAm-b-PPEGMA also showed a significantly higher rejection ratio with a longer ATRP polymerization time.

**Figure 6:**
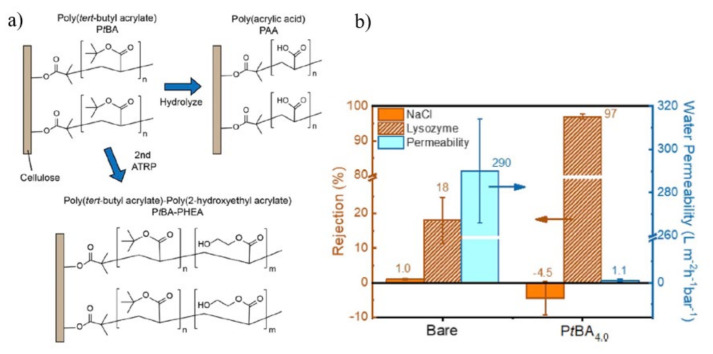
(**a**) Structure of polymer brush chains; (**b**) Permeability and rejection of pristine and modified membrane [123].

An ultrathin, mechanically durable and fouling-resistant coating of dense polymer brushes has been formed on cellulosic membranes [123]. The controlled grafting of polymer brush layers was accomplished through the employment of SI-ATRP reaction. After binding ethyl α-bromoisobutyrate (EBiB) initiator, with tert-butyl acrylate (tBA) and 2-hydroxy ethyl acrylate (HEA) as the monomers, p(tBA) chains were generated through homogenous SI-ATRP reaction as shown in Figure 6a. PtBA brushes hydrolyzed ester linkages and cleaved off brush layers, resulting in cleaved PAA chains. Ester linkages linked to tert-butyl sidechains were preferentially broken into carboxyl groups in dichloromethane with the presence of trifluoroacetic acid. As a result, a dense diblock copolymer of PtBA-PHEA layer was produced on the membrane surface. The grafting density of the modified membrane was not solely depending on ratios of initiator and cross-linker acyl halide content. Other factors, such as the binding rate of each kind of molecule and the steric hindrance of developing brushes, have significant influence on the resulting density. The grafted membrane exhibited enhanced hydrophilicity when compared to the pristine cellulose membrane. The rejection of lysozyme, a globular protein with a molar mass of 14.3 kDa, increased dramatically from 18 to 97% with the grafting of~4 kg mol^−1^ PtBA brush. However, the permeability dropped substantially from 290 to 1.1 Lm^−2^ h^−1^ bar^−1^, indicating that the membrane pores were completely covered as shown in Figure 6b. The presence of microscopic pores smaller than lysosomes was suggested by the fact that NaCl was not rejected before or after brush layer development. Tiny brush molecular weight may affect the flux, implying that SI-ATRP is useful in forming ultrathin composite membrane. 

**Figure 7:**
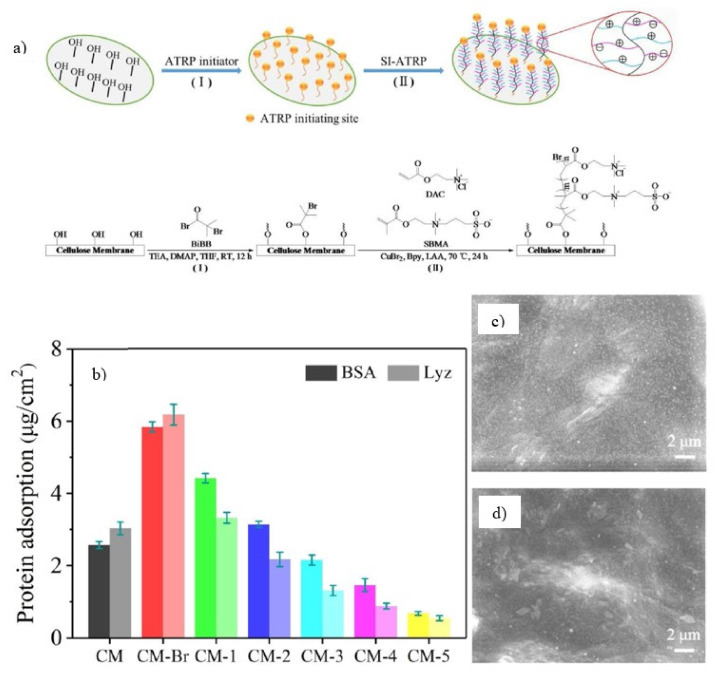
(**a**) Schematic illustration of SI-ATRP synthesis of zwitterion and quaternary ammonium copolymers from cellulose membrane; (**b**) Adsorption of BSA and Lyz on the cellulose membranes. SEM images represent the morphologies of BSA on the surface of (**c**) pristine cellulose membrane (CM); (**d**) modified CM-5 (CM = Unmodified membrane, CM (1–5) = Grafted membrane with various grafting degree and grafting ratio in which CM5 uses highest grafting degree and ratio) [118].

SI-ATRP was employed to graft zwitterionic copolymer brushes with different ratios of SBMA and [2-(Acryloyloxy) ethyl] trimethylammonium chloride (DAC) onto transparent cellulose membrane to improve its anti-fouling and antibacterial performance [118]. As illustrated in Figure 7a, the Si-ATRP initiator was first fixed to the membrane surface using α-bromoisobutyryl bromide (BiBB) to esterify hydroxyl groups on CM surface. Then, under the right circumstances (70 °C, 24 h), ATRP polymerized few random copolymer brushes of zwitterionic SBMA and cationic DAC (PSBMA-co-PDAC) and the equivalent homopolymer brushes from the grafted initiators. The anti-fouling characteristics of the cellulose membrane surfaces were evaluated using a static protein adsorption test at pH 7.4 using negatively charged BSA and positively charged Lyz as model proteins. As presented in Figure 7b, the adsorption of positively charged Lyz on the unmodified cellulose membrane was greater than that of negatively charged BSA, which can be attributed to their electrostatic responsiveness to the negatively charged BSA. Additionally, SEM images indicated high number of proteins covered on the pristine cellulose membrane (Figure 7c) compared to the considerably reduced coverage on the grafted membrane with highest grafting degree and ratio (Figure 7d), indicating that the zwitterionic surface can significantly hinder protein attachment. Zwitterions, in particular, can attach a high number of water molecules to produce a hydration layer on the material surface, creating a powerful repulsive force for protein at specified separation distances or making protein adsorption on the surface reversible without substantial conformational change [124].

**Figure 8:**
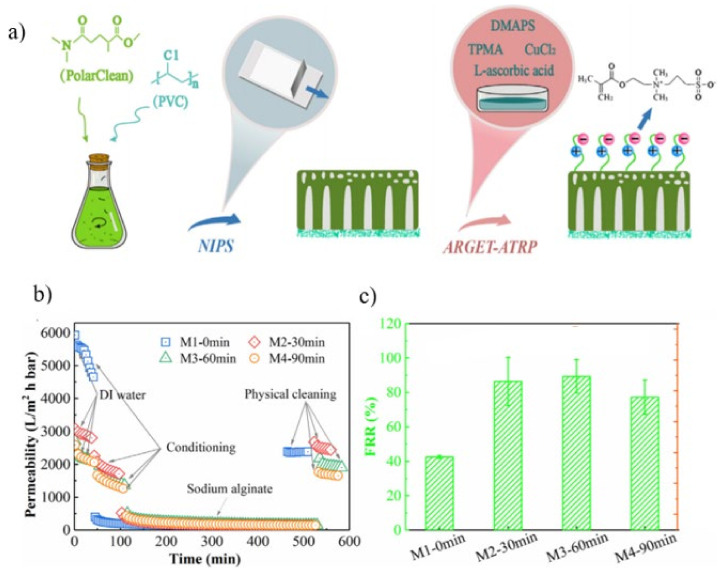
(**a**) Schematic overview of membrane fabrication where poly (vinyl chloride) PVC is dissolved in the green solvent PolarClean, followed by the production of a pristine membrane using the non-solvent phase-induced NIPS technique. Finally, using the ARGET ATRP technique, DMAPS polymers were grafted onto the membrane surface; (**b**) Permeabilities with time for M1-0 min, M2-30 min, M3-60 min, and M4-90 min; (**c**) FRR of M1–M4 [125]. M1 = Pristine membrane, M2 = Grafting time (30 min), M3 = Grafting time (60 min), M4 = Grafting time (90 min) [125].

Traditional solvents are used extensively in the non-solvent phase induced (NIPS) technique for UF membrane production. Methyl-5-(dimethylamino)-2-methyl-5-oxopentanoate, a green solvent, was used to fabricate poly (vinyl chloride) (PVC) UF membranes. Following that, a zwitterionic polymer, [2-(methacryloyloxy) ethyl] dimethyl- (3-sulfopropyl) ammonium hydroxide (DMAPS), was grafted onto the membrane surface using ARGET-ATRP reaction to improve its anti-fouling characteristics (Figure 8a) [125]. Surface grafting reduced the pure water permeability (PWP) of the membrane as the DMAPS brushes obstruct the pores on the membrane surface. The longer the grafting time, the greater the drop in PWP. Even though the PWP for grafted membranes was lower than that of pristine membrane, the values for all membranes were remained high in the range of 2121.8–2872.3 Lm^−2^ h^−1^ bar^−1^ for membrane with grafting duration of 30–90 mins (Figure 8b). Additionally, as shown in Figure 8c, the FRR rose substantially when zwitterionic DMAPS polymers were surface grafted. The development of a hydration layer around the zwitterionic DMAPS brushes, in which foulants were rejected by the polymer brushes owing to steric hindrance, was attributed to the rise in FRR after grafting. However, the anti-fouling property decreased by 77.2% when the grafting time was increased to 90 mins.

Corona air plasma graft copolymerization of zwitterionic monomer was grafted on the PES UF membrane to enhance the permeation flux of the membrane [126]. Peroxides were developed when corona discharge stimulated the membrane surface, and then decomposed into free radicals. The surface trapped metastable radicals served as initiators in the subsequent grafting processes. The activated membrane surfaces were immediately immersed in SBMA aqueous solution and the subsequent copolymerization was carried out at specified temperature (25 °C) for 0.5 to 30 h. The water contact angle of the treated membrane was reduced from 72° for unmodified PES membrane to 65°. The hydrophilicity of the modified membranes was slightly increased after zwiterrionization of the membrane surfaces. With increasing grafting temperature, the water contact angle of the modified membranes dropped gradually, confirming that hydrophilic groups-containing SBMA were effectively grafted onto the membrane surfaces. As a result, the water flux of the PES-g-SBMA membranes (579 Lm^−2^ h^−1^) was higher compared to corona treated (383 Lm^−2^ h^−1^) and pristine membrane (198 Lm^−2^ h^−1^). A maximum increase of nearly 1100% in permeate flow for oil/water emulsion filtering was obtained at a grafting yield of 0.489 mg/cm^2^. Furthermore, a maximum FRR enhancement of about 180% was obtained in PES-g-SBMA membranes compared to pristine membrane. This occurrence was ascribed to the development of a persistent hydration layer due to strong electrostatic hydrogen interactions between anionic and cationic species in the polymer chain and water molecules, which inhibited oil droplets in the feed from attaching to the surface.

Cellulose triacetate (CTA) FO membrane used for protein recovery was modified with two plasma gases, i.e., Ar and CO_2_, followed by AA grafting [127]. The greater hydrophilicity of AAc + Ar membranes was attributed to an inert characteristic of Ar gas, which creates more free polar radicals for reactions on the membrane surface than CO_2_, resulting in a greater water flux. The flux of AAc + Ar modified membrane was 8.12 Lm^−2^ h^−1^ and AAc + CO_2_ modified membrane was 7.56 Lm^−2^ h^−1^. Furthermore, reverse salt flux and fouling propensity were also reduced in a greater extent with the AA grafted membrane activated with Ar. Additionally, fouling studies using protein and polysaccharide showed that the AAc + Ar and AAc +CO_2_ modified membranes have an outstanding anti-protein fouling property, making them more suited for FO protein filtering. The modified membranes showed exceptional anti-protein fouling characteristics, making them ideal for protein recovery. The reversible fouling produced by BSA filtration for AAc + Ar membrane had the lowest R_re_ of 6.87 × 10^10^ m^−1^, which was 58% lower than the R_re_ of the pristine membrane. The increased hydrophilicity of the AAc + Ar membrane resulted in a lower Rre, since the membrane surface was less likely to absorb amphiphilic molecules like BSA [128]. The modified membrane exhibited higher selectivity and water flux but lower reverse salt flux and intrinsic membrane resistance (Rm) than the pristine membrane, suggesting that surface graft polymerization with Ar gas activation is an effective approach to enhance the overall performances of FO membranes. 

UV-initiated grafting polymerization of AA with silver containing metal–organic frameworks (Ag-MOFs) has been performed on UF membrane to enhance anti-fouling and antibacterial properties [129]. Benzophenone was used as the photo-initiator and AA was employed to increase surface hydrophilicity while offering sites for further Ag-MOF functionalization to accomplish the desired antibacterial and anti-fouling characteristics. The PWP of pristine membranes was in the range of 1500 - 2500 Lm^−1^ h^−1^ bar^−1^ while the modified membranes exhibited lower PWP of 1200 ± 260 Lm^−1^ h^−1^ bar^−1^, due to the extra barriers formed by AA or Ag-MOF layers. The Ag-MOF modified membranes exhibited *E. coli* and *S. aureus* inactivation rates of up to 90% and 95%, respectively. Silver ions are abundant in Ag-MOFs and it must be accessible to the microbial cell to achieve bacterial inactivation. MOFs’ three-dimensional structure may be tweaked to regulate and optimize the slow release of silver ions, preventing unwanted ion leaching from the membrane during the filtering process. The rate of silver release from the immobilized MOFs indicated that they were sufficiently immobilized and had long-term performance potential for the modified membrane. Table 2 summarizes the findings of performance evaluation for selected chemically-grafted integrally skin membranes.

### 4.2. Polyamide Thin Film Membrane

PA TFC membrane has three-layer configuration which provides high rejection of unwanted elements (such as salts), a high filtration rate, and superior mechanical strength [134]. The combined fabrication approach provides significant benefits as either the active layer or the substrate may be tuned individually to obtain desired membrane characteristics such as high flux/high rejection [135]. Vantanpour and Zoqi performed a chemical grafting on the surface of a PA TFC RO membrane with carboxylate multiwalled carbon nanotubes (COOHMWCNT)-augmented AA [136]. The grafting was mediated by an ethylene glycol dimethacrylate (EGDMA) cross-linker, with potassium persulfate (K_2_S_2_O_8_) and sodium metabisulfite serving as initiators in a redox system. The grafting conditions, such as the contact time and the curing period, exhibited a significant impact on the grafted membranes’ separation performance. The modified membranes with 3 mins of contact time and 80 mins of curing time in a 50 °C oven had the greatest flux and the lowest rejection decrease. The COOH-MWCNT and PAA significantly increased the membrane performance based on their hydrophilicity and fouling resistance. COOH-MWCNTs improved the yield of the polymerization by increasing the number of sites for AA monomer and increasing the amount of AA monomers bind to the membrane surface.

**Figure 9:**
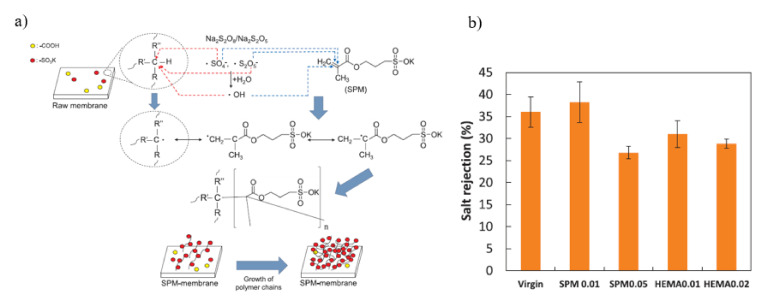
(**a**) Scheme of radical graft polymerization using 3-sulfopropyl methacrylate potassium salt (SPM) for membrane surface modification; (**b**) Salt rejection of pristine and SPM, hydroxyethyl methacrylate (HEMA) modified membrane with different concentrations [137]. Error bars represent one standard deviation of triplicate measurements.

Lin grafted 3-sulfopropyl methacrylate potassium salt (SPM) and 2-hydroxyethyl methacrylate (HEMA) on NF membrane using concentration-polymerization-enhanced radical graft polymerization technique to minimize severe silica fouling [137]. The radical graft polymerization approach for NF membrane surface modification using SPM is shown in Figure 9a. The contact angle of modified membrane reduced considerably compared to pristine membrane, demonstrating an increase in membrane surface hydrophilicity. The salt rejection of the modified membrane varied according to the monomer concentration. As shown in Figure 9b, the modified membrane with a low monomer concentration exhibited little change in NaCl rejection where the rejection was 38.2% for 0.01 M SPM, 31.0% for 0.01 M HEMA and 36.1% for the pristine membrane. On the other hand, the membrane grafted using a greater monomer concentration showed a substantial reduction in salt rejection where the rejection was 26.8% for 0.05 M SPM and 28.8% for 0.02 M HEMA. The monomer and initiator may penetrate into the supporting layer during filtration and resulted in membrane hydrolysis and partial degradation which subsequently increased the permeability at the expense of the NaCl rejection. Additionally, the primary silica fouling mechanism shifted from gel layer development to intermediate or full blockage, implying that fewer silica particles were formed on the grafted membrane. Modified NF membrane with low SPM or HEMA concentrations is advantageous for silica fouling mitigation.

**Figure 10:**
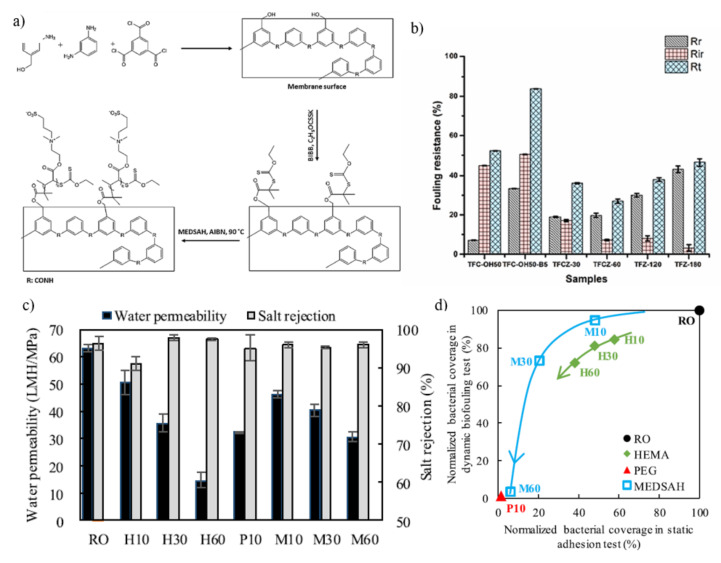
(**a**) Grafting of ((3-methacryloylamino) propyl) dimethyl(3-sulfopropyl) ammonium hydroxide (pMEDSAH) onto PA) thin film composite (TFC) nanofiltration (NF) membrane via surface-initiated RAFT polymerization; (**b**) Fouling resistance ratio of the prepared NF membranes. The BIBB-stabilized and pMEDSAH grafted membranes are referred as TFC-OHi-Bj and TFCZ-t, where i, j, and t refer to the DAPM (wt.%), BIBB (wt.%), and polymerization time (minute); (**c**) Water permeability and salt rejection of pHEMA-, pPEG-, and pMEDSAH-grafted membranes with different polymerization durations; (**d**) Relationship between normalized bacterial coverage in static bacterial adhesion and in dynamic biofouling filtration [140]. The direction of arrows means the increase of polymerization time.

Nadizadeh & Mahdavi grafted poly[2-(methacryloyloxy)ethyldimethyl-(3-sulfopropyl) ammonium hydroxide] pMEDSAH onto PA TFC NF membrane via SI-RAFT polymerization to improve surface hydrophilicity as shown in Figure 10a. The water contact angle increased from 74 ± 2.7° to 91 ± 0.4° upon the introduction of the azobisisobutyronitrile (AIBN) initiator on the membrane surface but was lowered following pMEDSAH grafting. The hydrophilicity of the NF membrane surface improved as the polymerization time progressed due to the zwitterionic polymer’s high hydration capacity. Due to the significant hydration capacity of the zwitterionic polymer, the hydrophilicity of the NF membrane surface increased as the polymerization time increased [138]. The grafted TFC membrane with grafting duration of 120 mins exhibited the optimal performance in terms of salt and dyes rejection (salt:36.24 ± 0.1%, dyes > 96%), water flux (1.45 ± 0.1 Lm^−2^ h^−1^ bar^−1^) and flux recovery ratio (92 ± 1.3%). The reversible fouling (Rr) ratio, irreversible fouling (Rir), and total fouling ratio (Rt) for the constructed membranes are shown in Figure 10b. Rir was also reduced significantly, from 45 ± 0.1% for the membrane with 50 wt.% of DAPM to 3.2 ± 1.6% for the pMEDSAH grafted membrane with polymerization time of 180 min. Due to the rapid efficiency recovery upon cleaning, which was induced by the formation of protein repulsive hydration on the membrane surface, the pMEDSAH grafted membranes exhibited a better fouling resistance than the BIBB-stabilized membranes.

Yang and colleagues grafted PA RO membranes with pHEMA, pPEG and pMEDSAH using SI-ATRP to enhance the biofouling resistance of the modified membrane [139]. As shown in Figure 10c, water permeability clearly reduced with increasing polymerization time due to the formation of thick pHEMA and pPEG layers. In particular, as compared to the pristine RO membrane, the grafting of pPEG membrane for 10 mins experienced 50% reduction in water permeability. However, the grafted pHEMA, pPEG, and pMEDSAH layer had no substantial influence on salt rejection when compared to the pristine RO membrane. The relationship between polymerization time and normalized bacterial coverage is shown in Figure 10d. All pHEMA-, pPEG-, and pMEDSAH-grafted membrane surfaces showed lower bacterial coverage compared to that of pristine RO membrane surface. In the static bacterial adhesion test, the normalized bacterial coverage of pHEMA- and pMEDSAH-grafted membranes decreased from 58% to 38% and 48% to 6%, respectively, when grafting period was increased from 10 to 60 mins. In addition, normalized bacterial coverage of pHEMA- and pMEDSAH-grafted membranes reduced from 84% to 72% and 95% to 3% in the dynamic biofouling filtration test, respectively. The normalized bacterial coverage of pPEG grafted membranes with a 10-min polymerization time was under 1% in static and dynamic biofouling experiments. The work summarized that the SI-ATRP polymerization time should be carefully regulated to control the main chain length of the grafted pHEMA and pMEDSAH, to optimize the anti-bacterial efficiency of the modified membrane.

**Figure 11:**
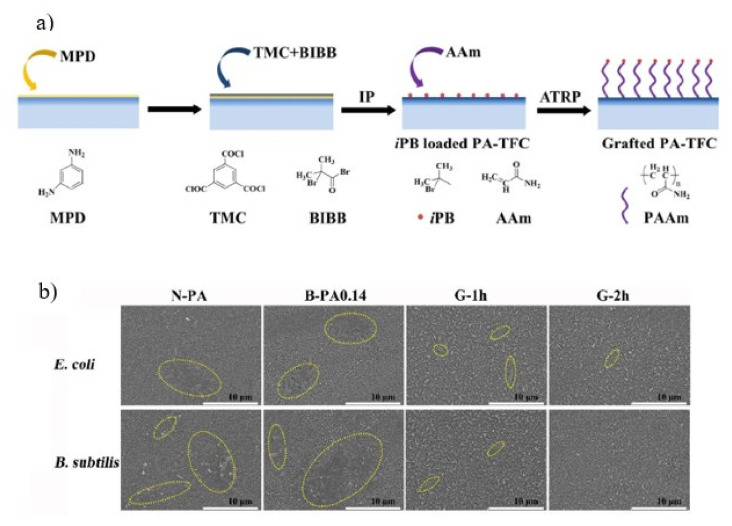
(**a**) Schematic illustration of membrane fabrication using PAAm by ATRP; (**b**) SEM images of N-PA, B-PA0.14, and grafted membranes after immersion in *E. coli* and *B. subtilis* suspensions for 24 h. The circles indicate the observed bacteria [140].

Zhao and his colleagues employed an in-situ ATRP method to graft polyacrylamide (PAAm) onto the PA-TFC to improve the biofouling resistance of RO membranes [140]. The ATRP initiators, isobutyryl bromide (iPB), were incorporated into the PA matrix using a bifunctional small molecule, 2-bromoisobutyryl bromide (BIBB), which was added in organic hexane solutions, as illustrated in Figure 11a. The fabricated RO membranes were labelled N-PA (nascent PA in n-hexane solution with no BIBB) and B-PA0.14 (nascent PA in n-hexane solution with BIBB). PAAm grafted membranes show a minor progressive reduction in water permeance and barely changed salt rejections when compared to pristine RO membranes. Water permeance and salt rejection for pristine membranes were 2.5 Lm^−2^ h^−1^ bar^−1^ and 99.2%, whereas for PAAm grafted PA membranes are 2.4 Lm^−2^ h^−1^ bar^−1^ and 99.0%, respectively [140]. More notably, the grafted membranes showed synergistic anti-adhesion and bacteriostatic efficacy against *E. coli* and *B. subtilis* bacteria. The increased hydrophilic surface resulting from the ATRP grafting of PAAm inhibits bacterial adsorption and/or deposition on the membrane surface, which is primarily attributable to the hydration layer on the membrane surface. Furthermore, the reduced surface roughness also helped in improving the anti-adhesion properties of the modified membrane. In the dynamic BSA fouling test, the grafted membranes had a lower final flux decline ratio (FDRf) and a higher final FRR than the pristine membranes. On the N-PA0.14 and B-PA0.14 surfaces, a substantial mass of *E. coli* or *B. subtilis* colonies was observed. However, as shown in Figure 11b, only a small number of bacteria was observed on the membrane surfaces grafted for 1 h and 2 h, showing that PAAm grafting is effective in inhibiting bacteria attachment. 

**Figure 12:**
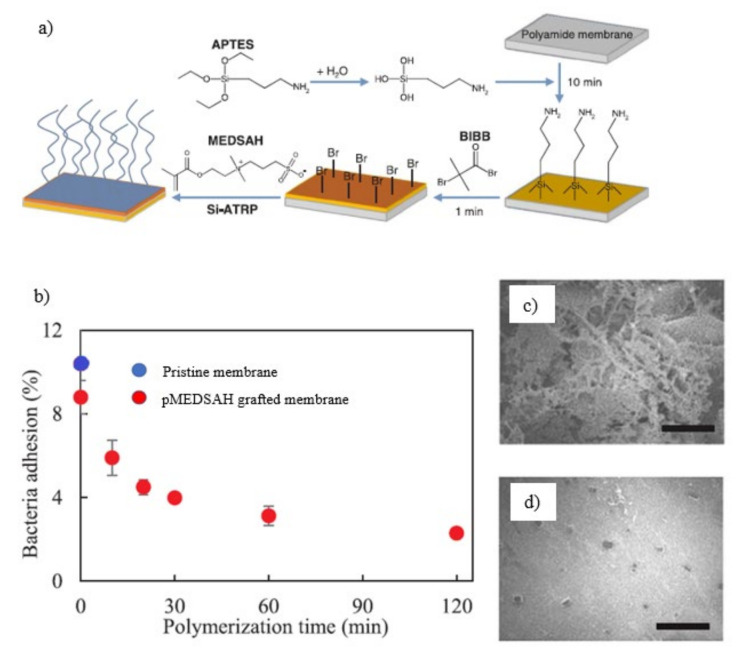
(**a**) Schematic illustration of SI-ATRP reaction of MEDSAH on a PA.RO membrane after APTES pretreatment; (**b**) Bacterial adhesion on pristine and pMEDSAH- grafted membranes at different polymerization times. FESEM images of bacterial adherence during dynamic biofouling filtration tests on the surface of (**c**) Pristine RO membrane; (**d**) pMEDSAH-grafted membrane at polymerization time of 120 min [141].

Yang and colleague grafted zwitterionic pMEDSAH on the surface of BIBB-immobilized PA RO membranes through SI-ATRP [141]. As shown in Figure 12a, the surface of RO membranes was first aminated with 3-aminopropyltrimethoxysilane (APTES). The APTES layer was then treated with -bromoisobutyryl bromide (BIBB), an acyl halide-type ATRP initiator. The membrane surface was then grafted with a zwitterionic polymer, pMEDSAH, through SI-ATRP. The grafting of pMEDSAH improved the surface hydrophilicity and resulted in a smoother surface as the polymerization time increased. However, water permeability reduced significantly as the polymerization time increased, due to the thick coating of pMEDSAH forming on the membrane surface, obstructing water penetration. Salt rejection scarcely changed after pMEDSAH grafting. The rejection for modified membrane with polymerization time of 120 mins was 94.5% while the rejection for pristine membrane was 96%. In terms of biofouling, pMEDSAH-grafting effectively inhibited bacterial adherence to 2.0% from 9.5%, which decreased as the polymerization time increased as in Figure 12b. As in Figure 12c, the surface of the pristine membrane was substantially covered in bacteria, suggesting that bacteria quickly adhered to the surface of the pristine membrane and developed a biofilm. The biofilm grows smaller, thinner, and less thick as the polymerization time was increased up to 120 mins for the modified membrane (Figure 12d). The dense and smooth grafted membrane led to superior biofouling resistance as the reduced surface roughness decreased the surface area for membrane–foulant interaction. 

**Figure 13:**
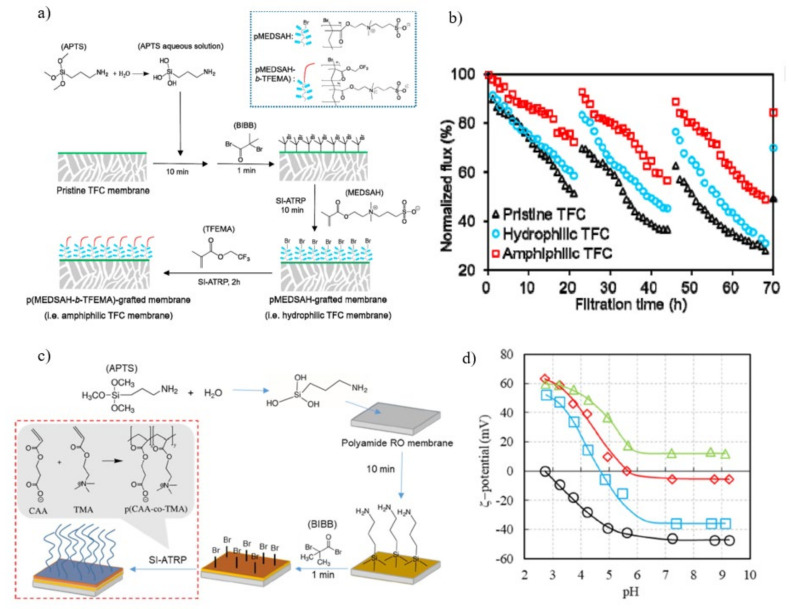
(**a**) Schematic illustration for the amphiphilic p(MEDSAH-*b*-TFEMA)-grafted membrane fabrication through dual SI-ATRP; (**b**) The flux of pure TFC, hydrophilic TFC, and amphiphilic TFC membranes was shown as a function of filtration time during biofouling [142]; (**c**) Schematic illustration of the fabrication of the p(CAA-co-TMA)-grafted membranes by the SI-ATRP method; (**d**) Zeta-potential of membrane as a function of pH (Black = RO, Blue = CAA:TMA ratio of 6, Red = CAA:TMA ratio of 3, Green = CAA:TMA ratio of 1) [145].

Yang and group utilized SI-ATRP to sequentially graft two materials onto TFC RO membrane surfaces i.e, pMEDSAH with strong hydrophilicity, and poly (2,2,2-trifluoroethyl methacrylate) (pTFEMA) with low surface energy [142]. As in Figure 13a, pristine TFC RO membrane was immersed in a 20 mL 1 *v*/*v*% APTS aqueous solution for 10 mins before being transferred to a 10 mL hexane solution containing 3 wt.% BIBB for 1 min. The membrane is initially grafted with pMEDSAH for 10 mins using SI-ATRP. The pMEDSAH-grafted membrane was next grafted with TFEMA for 2 h, resulting in the formation of p(MEDSAH-b-TFEMA)-grafted membranes. The water permeability of the hydrophilic pMEDSAH-grafted and amphiphilic p(MEDSAH-b-TFEMA)- grafted TFC membrane was 3.5 Lm^−2^ h^−1^ bar^−1^ and 3.0 respectively. It shows a slight decrease in permeability when compared to pristine membrane (5.3 Lm^−2^ h^−1^ bar^−1^). The reduction in water permeability was caused by a higher hydraulic resistance resulted from the surface modification. As shown in Figure 13b, the pristine TFC membrane reported the steepest water flow reductions, falling to 51%, 37%, and 28% of its original water flux during the three cycles due to its comparatively hydrophobic surface. The hydrophilic pMEDSAH-grafted membrane on the other hand, showed gentler water flow reductions of 58% and 45% for the first and second cycles respectively, demonstrating the anti-fouling capabilities of hydrophilic modifications. Unfortunately, the hydrophilic TFC membrane’s water flux decreased with time, and by the end of the third cycle, it was only 31% of its original water flux, which was comparable to that of the pristine TFC membrane. The amphiphilic TFC membrane, exhibited outstanding anti-fouling capabilities against bacteria, as seen by lower water flux decreases (72%, 57%, and 49% for the three biofouling cycles, respectively) than the other two membranes. The hydrophilic membrane surface is typically subjected to more concentrated foulants than those present in the bulk due to foulant advection toward the membrane by the permeate flow and foulant rejection by the active layer. As a result of the poor hydration layer caused by the attached foulants on the membrane surfaces, the anti-fouling characteristics of hydrophilic membranes may gradually deteriorate. However, the fouling resistant properties of pMEDSAH significantly alleviated water flux decline while the fouling release properties of p(MEDSAH-b-TFEMA) render foulant attachment unstable, further reducing the water flux decline and improving water flux recovery during dynamic filtration [143,144]. 

To improve its anti-biofouling properties, a PA RO membrane was modified with a polyampholyte composed of anionic 2-carboxyethyl acrylate (CAA) and cationic [2-(acryloyloxy) ethyl] trimethyl ammonium chloride (TMA) through SI-ATRP [145]. As shown in Figure 13c, mixed monomer aqueous solution containing CAA and TMA in a specific molar ratio was prepared. Amination with 1 *v*/*v*% APTS occurred on a PA RO membrane for 10 mins followed by immobilization with 3 wt.% BIBB for 1 min. Later, CAA/TMA polyampholytes were grafted on the surface of PA RO membranes using the SI-ATRP process. The water permeability of the modified membrane decreased as compared to that of pristine membrane (60 Lm^−2^ h^−1^/MPa) but increased as the TMA portion of the modified layer increases. Salt rejection of the modified membrane (80%) was lower than that of pristine membrane (99%) and decreased as the TMA portion increased. The reduction in water permeability of the modified membrane was due to increased surface layer thickness. Additionally, with increasing amounts of TMA in the polymerization solution, the isoelectric point (IEP) of the p(CAA-co-TMA)-grafted membranes progressively moved to a higher pH, suggesting that surface positivity increased as shown in Figure 13d. With the increasing pH, the zeta potential of the p(CAA-co-TMA)-grafted membranes changed from positive to negative charge, and remained almost constant above pH 7. Furthermore, biofouling occurred even for a membrane surface with a high hydrophilicity when there was a significant electrostatic interaction between the membrane surface and the foulants [146]. Contrarily, in the absence of electrostatic interaction between the membrane surface and foulants, the anti-biofouling capabilities of the membrane were governed by its hydrophilicity. It was revealed that the modified RO membrane with a 1:1 CAA/TMA surface ratio, manufactured from a mixed monomer solution with a 3:1 CAA/TMA ratio, had almost negligible net surface charge above pH 6 and performed the best in the long-term dynamic biofouling test.

Liu and colleagues demonstrated the anchoring of poly[1-vinyl-3(2-carboxyethyl) imidazolium betaine] (PVCIB) brush onto a commercial PA TFC FO membrane [31] by first grafting polyvinyl imidazole (PVI) via 2,2-azobis(isobutyramidine) dihydrochloride (AIBA)-initiated free radical graft polymerization and followed by betanization using 3-bromopropionic acid as the quartenizing agent. The electron donor nitrogen of the imidazole ring was quaternate throughout the process, resulting in brushes that were covalently bonded to the PA surface. The presence of grafted PVI increased membrane hydrophilicity due to the water solubility and propensity of PVI to be partly protonated. As a result, the water flow of the PVCIB FO membrane increased to 91.6 Lm^−2^ h^−1^, an increase of nearly 25% in relative to that of pristine membrane. The PVCIB-modified membrane also displayed 98.8% of *E. coli* mortality rate due to the capacity of PVCIB to limit bacterial growth by altering bacterial cellular metabolism. 

Membrane surface charge has a remarkable impact on the ion transport selectivity as well as fouling processes in water treatment. TFC RO membrane was plasma-polymerized with maleic anhydride (MA), VIM and the membrane’s surface charge were well customized [147]. With increasing amine groups on the surface after plasma polymerization with VIM monomer, negatively charged control membranes gained positive charges. In contrast, plasma polymerization with MA has dramatically increased the density of negative charges as the density of carboxylic groups increased. The water flux of the unmodified membrane was 44.9 Lm^−2^ h^−1^. However, plasma treatment with VIM resulted in a considerable improvement, as the water flux increased to 47.2 Lm^−2^ h^−1^ and 49.2 Lm^−2^ h^−1^ after 5 and 9 mins of treatment, respectively. The flux values plummeted to 44.2 Lm^−2^ h^−1^ when the treatment duration was increased beyond 9 mins, which was ascribed to the membranes’ high mass transfer resistance as a result of the thicker and denser polymerized layer deposit. The NaCl rejection was sustained around 97% throughout the process. 

**Figure 14:**
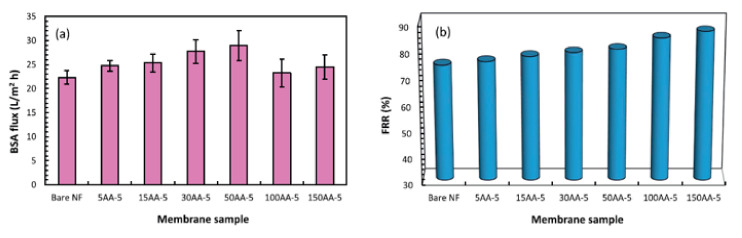
(**a**) BSA flux; (**b**) FRR of the AA-grafted NF membranes with various AA concentrations [148].

Vatanpour and colleagues performed UV-initiated grafting on PA TFC NF membrane with 50 g/L AA as the optimal sample due to its salt rejection and anti-fouling performance [148]. The more hydrophilic polyacrylic acid layer develops on the membrane surface as a result of the grafting process, increasing the membrane’s permeability. However, long UV exposure up to 10 min thickened the grafted layer and limited membrane permeability. The enhanced hydrophilicity of these membranes was confirmed by contact angle analysis. When the UV duration was raised to 5 mins, the rejection of both divalent (Na_2_SO_4_) and monovalent (NaCl) salts increased, and when the UV duration was raised to 10 mins, the rejection values remained nearly constant. The growth of the generated grafting layer enhanced the surface negative charge and facilitates salt rejection by increasing the UV time. By increasing the grafting monomer concentration up to 50 g/L, the BSA flux of the modified membranes gradually elevated, as shown in Figure 14a. Further increases in monomer concentration resulted in a decrease in protein flow, due to the thickening of the grafting layer. All modified membranes exhibited a greater FRR value than the pristine NF membrane, as shown in Figure 14b. Due to the reduced surface roughness accompanied with increased surface negative charge and surface hydrophilicity, the modified membranes demonstrated improved permeability, salt rejection, and anti-fouling ability.

Commercial PES NF membrane was modified via UV grafting using AA and used in FO application [149]. The UV irradiation was initiated when the phenoxyphenyl sulfone chromophores in the PES chain absorbed UV light. Homolytic cleavage occurred at the carbon-sulfur bond in sulfone linkage. Two radical sites were created at the polymer chains as a consequence of this action. The free radicals then react with the monomer solution (AA) to produce the carboxyl group of the polymer trunk’s chain. The reduction in water contact angle for the modified membrane showed that surface modification via UV grafting enhanced the surface hydrophilicity. Modified membranes showed a four-fold greater water flux compared to pristine membranes. The water flux of the pristine membrane was 0.75 Lm^−2^ h^−1^. At the lowest monomer concentration of 5 g/L AA and grafting time of 1 min, water flux increases dramatically to 2 Lm^−2^ h^−1^ due to the development of a carboxylic group on the membrane surface aided water molecule movement. In comparison to lower monomer concentrations and lower monomer time, monomer concentrations of 30 and 50 g/L AA at 3 and 5 min exhibited decreasing flux values. At increasing monomer concentrations and grafting time, the improved grafted layer on the membrane surface reduced water flux. The thickening of the grafted layer as a result of an effective and increased grafting duration on the membrane surface improved flux resistance. The graft polymerization involved two competitive mechanisms, i.e., efficient grafting and chain scission. The effective grafting outperformed chain scission for 30 g/L AA grafted for 1 min, resulted in salt rejection of 53%. However, chain scission dominated the graft polymerization for 50 g/L AA grafted for 1 min, resulted in salt rejection of 22%. The salt rejection value also fluctuated more at higher monomer concentrations (30 and 50 g/L), where the pH value is less than 3. Therefore, it is recommended to utilize a lower monomer concentration of AA since this creates an appropriate environment for a more effective grafting.

**Figure 15:**
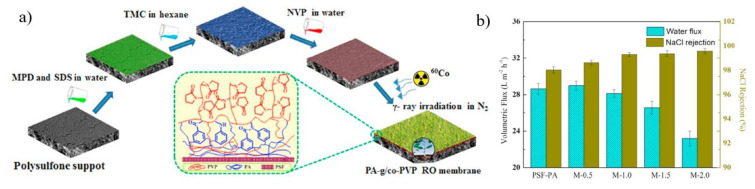
(**a**) Schematic overview of fabrication process for PA-g/co-PVP RO membranes via multistep interfacial polymerization; (**b**) Volumetric flux and salt rejections of nascent membranes PSF-PA and PA-g/co-PVP RO membranes M-0.5, M-1.0, M-1.5, and M-2.0 (cross-flow velocity 0.19 m/s; 2000 ppm NaCl feed solution; 25 °C; pH 7.0; and TMP15.5 bar) (M-0.5 to M-2.0 shows various PVP loading, where M-0.5: 0.5 kg/m^3^ PVP, M-1.0: 1.0 kg/m^3^ PVP, M-1.5: 1.5 kg/m^3^ PVP, M-2.0: 2.0 kg/m^3^ PVP) [150].

A novel PA-g/co-PVP RO composite membrane was grafted with N-vinyl-2-pyrrolidone (NVP), polyvinylpyrrolidone (PVP) and gamma irradiated using Cobalt-60 for pharmaceutical wastewater treatment [150]. As in Figure 15a, RO membrane was generated on the PSF support membrane through IP. An aqueous solution containing MPD, SDS and TMC in n-hexane was placed over the PSF membrane’s surface. Next, a solution containing NVP used as a “seed agent” that was invaded into a low cross-linked PSF-PA surface at a concentration of 0.5 to 2.0 kg/m^3^. Later, a hydrophilic molecular brush of polyvinylpyrrolidone (PVP) was grafted on the PA surface. The membrane was irradiated with a Cobalt-60 gamma source. The surface hydrophilicity of the modified membranes improved considerably following PVP grafting. Nevertheless, as shown in Figure 15b, the volumetric flux of the PA-g/co-PVP RO membrane decreased constantly as the NVP concentration increased. The greater intermolecular hydrogen bonding and increased hydraulic resistance resulted in a drop in PA-g/co-PVP RO membrane flux, which in turn led in a flux reduction. However, the modified membrane’s salt rejection improved significantly, showing that the functional structure of the PA active layer was completely under control and remained intact during the alteration. Moreover, the PA-g/co-PVP RO membrane has superior anti-fouling efficacy against BSA, SA, and SDS aqueous solutions when compared to the nascent membrane. The pristine membrane PSF-PA exhibited FRR of 62.28%, 86.92%, and 82.81% for BSA, SA, and SDS, accordingly, whereas the PA-g/co-PVP RO membrane with 1.0 kg/m^3^ PVP loading (M-1.0) produced higher FRR values of 91.23%, 96.28%, and 93.67% for the foulants of BSA, SA and SDS, accordingly. Table 3 shows the performance evaluation of modified and pristine membrane on various approaches such as chemical, plasma and radiation-initiated grafting discussed in this section.

## 5. Challenges and Future Direction

Membrane modification through graft polymerization is a potential method to enhance membrane hydrophilicity, anti-fouling and anti-chlorine properties. Through the anchoring of hydrophilic polymer chains on the membrane surface, graft polymerization enhances the hydrophilicity of membranes and improves fouling resistance. Similarly, chlorine-capture functional groups have also been chemically grafted to render chlorine resistance. Over the last decade, substantial efforts have been made in the development of chemically grafted liquid separation membranes. Despite the versatility of graft-polymerization in heightening the performances of liquid separation membranes, some technical issues remain great challenges for the practical applications in large-scale membrane processes.

Enhancing membrane permeability while preserving salt rejection capability is important when developing a new membrane. One significant challenge in graft polymerization is the reduction of water flux upon the introduction of grafting layer [159]. Although highly hydrophilic materials have the potential to minimize the degree of water flow reduction, hydrophilicity is often inadequate to offset the negative impacts of the additional transport resistance introduced by the grafting layer. Additionally, even though modified membranes are less prone to fouling, total flux is always lower upon the graft-polymerization modification. Besides imposing transport resistance, the blockage of membrane pores by the grafted polymer chains is another reason for the loss of membrane flux. Grafting of polymers with high chain density and molecular weight further increases flux loss although the grafted chains offer the requisite surface hydrophilicity to reduce membrane fouling. Therefore, striking a balance between flux and membrane surface functionality introduced through graft polymerization is a major concern when implementing graft polymerization for membrane surface modification. Selecting appropriate materials along with careful planning of the reaction condition are an efficient way to achieve the desired surface modification outcome.

Most graft-polymerization processes consist of multi-step reactions, which takes a long time to accomplish the modification. The simplicity of the experimental procedures and labor savings can greatly reduce handling costs, which is an appealing motivator for bringing the notion of surface functionalization to the industrial level. With many steps and parameters involved in the grafting process, optimization of the grafting parameters is important to maximize the benefits of the modification. Chemical surface modification can be optimized in a variety of ways. The concentration of monomers is still a crucial metric to be taken into consideration. The orientation and conduct of functional moieties or polymer chains on the membrane surface might differ dramatically with their concentration variations. For example, at low concentrations, polymer chains may form an interlaced structure, but at high loading, they transform into a polymer brush with a great degree of spatial flexibility. The orientation can alter the physio-chemical characteristics of the grafted surface, which in turn affects the permeability and rejection of the membranes. The use of optimization tool like response surface methodology (RSM) optimization is common in graft polymerization. It helps to optimize parameters by providing fast and accurate experimental data and by their capacity to evaluate the effects and interactions between variables effectively [160]. With the aid of RSM design, the parameters of the experiment such as UV-activation time and solvent concentrations can be studied to determine their relationship with the responses like permeability and rejection. A quadratic model will be created for each of the parameters and responses. In addition, the model will be tested using analysis of variance (ANOVA) and the model can accurately estimate the most desired optimal point for a process [161].

Chemical grafting usually requires a lot of chemicals which are generally too costly to justify large-scale manufacturing. Chemical initiators such as BPO, AIBN, and K_2_S_2_O_8_ are required to create free radicals during the polymerization process [162]. Most of these initiators are hazardous and ecologically unfriendly. Lately, increasing efforts have been made in exploring more environmentally and cost friendly approaches. Green chemistry is one of such strategies that can offer long-term solutions for more sustainable surface modification [151]. Monoterpenes (MTs), ionic liquids (ILs), and supercritical fluids (CO_2_) are among the recommended solvents for graft polymerization. These eco-friendly substitute solvents are equivalent to traditional fossil fuels in terms of performance. For instance, free-radical polymerizations can be carried out in ILs, with the molecular weights and rates of reactions being found to be greater than in organic solvents [163]. Supercritical fluids with gas-like diffusivities are advantageous for reactions and their liquid-like densities can allow the solvation of a wide range of molecules. As solvents have distinct effects membrane morphologies and properties through their interaction with the membrane surface, it is important identify more new solvents and investigate the suitability of solvent replacement in the grafting processes. 

The reactions involved in graft polymerization has some inherent issues. Catalyst residue is a common problem in graft polymerization. The techniques for dealing with catalyst residue are still in progress. For large-scale industrial activities, cost-effective catalyst separation techniques are required. The catalysts generally co-precipitate in the polymers as catalyst residues, which change the color of the membrane and may render them toxicity [164]. As a result, for most applications, the catalyst residue must be kept minimum, for safety reasons. One of the efficient ways in mitigating catalyst residue is by using a novel organic semiconductor graphitic carbon nitride (C_3_N_4_) catalyst in polymer brushes for surface-initiated photo atom transfer radical polymerization (SI-photo ATRP) [165]. It has facile preparation, acceptable bandgap (2.7 eV), excellent stability, low cost, visible light response, and is non-toxic. This catalyst has been widely used as photocatalyst for radical polymerization. Besides, g-C_3_N_4_ catalyzed SI-photo ATRP is temporal and spatial control. It can address the catalyst residues problem as it is a heterogeneous reaction system. Addition of C_3_N_4_ catalyst grafted with a polymer shows significant improvement in terms of hydrophilicity and anti-fouling property. In addition, g-C_3_N_4_ is easier to modify to improve the photocatalyst property. Thus, the heterogeneous reaction system of g-C_3_N_4_ catalyzed SI-photo ATRP offers a huge potential for graft polymerization of membrane surface modification.

Computational modelling of membrane surface modification has been presented as a technique to get understanding of how modifying chemicals interact with the membrane surface [166]. However, computational modelling has not been studied in depth in chemical grafting. With a better understanding of the mechanisms through simulation or modelling studies, the operating conditions involved in the chemical grafting can be fine-tuned more systematically. Notably, artificial intelligence technology can serve as a significant tool for surface modification of membrane [167]. Data-driven techniques, such as machine learning (ML), have been investigated to provide details on nanocomposite membrane manufacturing and modification, critical elements that influence membrane performance, as well as to forecast membrane attributes. For instance, ML uses multivariate analysis to reveal intricate connections between polymerization results and conditions, allowing it to prescribe the best reaction conditions for achieving discretionary polymer objectives. Besides, ML can be utilized to predict the grafting yield in radiation-induced graft polymerization reactions. It can also help to determine the accuracy of grafting yield prediction and the relevance of parameters of monomers [168].

## 6. Concluding Remark

This review focuses on chemical grafting modification methods for membrane processes and the applications to close this gap where the accomplishments of the last five years in this field are highlighted. In parallel to the rise of research efforts in various applications, notably wastewater treatment and desalination, an exponential surge in papers has been observed in grafting-from technique for membrane modification. This review establishes a foundation for the chemical grafting method, particularly the on grafting-from technique based on previous work. To date, substantial efforts have been made in chemical grafting modification methods for membrane surfaces to attain the desired membrane structural properties and separation performances. The benefits and drawbacks of several grafting-from chemical surface modification techniques for membrane processes are explored. It is hoped that this review could present some fascinating options and serve as a blueprint for the development of sustainable membranes for desalination and wastewater treatment by analyzing different chemically grafting oriented approaches that may be effectively employed in membrane modification.

## Figures and Tables

**Table 1:** Graft polymerization of polymeric membranes.

Grafting Method	Membrane Material	Polymer/Additive	Induction	Characteristic Introduced to the Modified Surface	Ref.
Chemical-induced	PP	HEMA	Ozone	The HEMA grafted PP membrane surface became hydrophilic and less adsorbable to bovine serum albumin (BSA) proteins compared to pristine membrane.	[71]
	PES	Poly (ethylene glycol) methacrylate (PEGMA)	Peroxydisulfate, Metabisulfite	The modified PES membrane showed additional absorption bands in the area of aliphatic stretching vibration, which was missing from the original membrane’s spectra.	[110]
Plasma-induced	PP	TiO_2_	Plasma: Air, O_2_	Due to the increased hydrophilicity, modified membranes demonstrated greater resistance to protein fouling as compared to pristine membranes.	[111]
	PES	AA	Plasma: Argon	The modified membranes are less susceptible to protein fouling than the pristine membranes, and plasma treatment greatly improved the modified membrane’s water flux. The modified membranes can be cleaned more easily and use less caustic to recover permeation flux.	[112]
Irradiation-induced	PP	PEGMA, HEMA	UV	The grafted pHEMA and PEGMA surface shows tremendous increase in pure water flux while substantially reducing protein adsorptions.	[113]
	PES	AA, Ethylene diamine, HEMA	UV	UV photo-grafting of hydrophilic monomers onto the membrane surface greatly improved the hydrophilicities of the membranes.	[114]
	PAS	Methacrylic acid, Glycidylmethacrylate (GMA), HEMA	UV	Modified membrane exhibited enhanced hydrophilicity compared to unmodified membrane.	[115]
	PVDF	PES	UV	BSA adsorption was reduced in the modified membrane, and flux recovery was improved.	[116]

**Table 2:** Performance evaluation of modified and pristine asymmetric integrally skin membranes.

Approach	Modification Materials/Membrane Material	Process	Surface—Grafted Membrane	Pristine Membrane	Ref.
SI-ATRP	PAA	UF	Rejection: 92% Flux: 230 Lm^−2^ h^−1^	Rejection: 90% Flux: 310 Lm^−2^ h^−1^	[122]
SI-ATRP	PNIPAAm PEGMA	UF	FRR: 99.1% Oil rejection: 98.2%	FRR: 97.1% Oil rejection: 91.1%	[119]
SI-ATRP	PAA PtBA-PHEA copolymer	Cellulosic membrane	Lysozyme rejection: 97% Permeability: 1.1 Lm^−2^ h^−1^ bar^−1^	Lysozyme rejection: 18% Permeability: 290 Lm^−2^ h^−1^ bar^−1^	[123]
SI-ATRP	SBMA DAC	Cellulose membrane	CFU Reduction *S. aureus*: 95.1% *E. coli*: 90.5%	CFU Reduction: *S. aureus*: 9.7% *E. coli*: 7.2%	[118]
ARGET-ATRP	DMAPS Zwitterionic	UF	Permeability: 2872.3 Lm^−2^ h^−1^ bar^−1^ FRR: 86.4% Rejection: 96%	Permeability: 500.0 Lm^−2^ h^−1^ bar^−1^ FRR: 42.6 ± 0.9% Rejection: 93.2 ± 2.4%	[125]
AGET-ATRP	HEMA	UF	Flux: 752.5 Lm^−2^ h^−1^ bar^−1^	-	[130]
Plasma initiated grafting	SBMA zwitterionic monomers Corona air	UF	Flux: 800 Lm^−2^ h^−1^	Flux: 198 Lm^−2^ h^−1^	[126]
Plasma initiated grafting	Ar/O_2_ plasma assisted oxygen activation in PSF membrane	UF	Flux: 350.7 Lm^−2^ h^−1^ Fouling resistance: 82% BSA Rejection: 99.9%	Flux: 25.2 Lm^−2^ h^−1^ Fouling resistance: 50% BSA Rejection: 55%	[131]
Plasma initiated grafting	CMB	UF	BSA Adsorption: 0.088 mg cm^−2^ grafted CMB: 0.045 mg cm^−2^ 0.023 mg cm^−2^ grafted CMB: 0.023 mg cm^−2^	BSA Adsorption: 0.096 mg cm^−2^	[132]
Plasma initiated grafting	AA with Ar and CO_2_	CTA	Flux_Ar_: 8.12 Lm^−2^ h^−1^ Flux_CO2_: 7.56 Lm^−2^ h^−1^	Flux: 6.11 Lm^−2^ h^−1^	[127]
UV initiated grafting	Zwitterionic acrylate monomer	UF	BSA Adsorption: 50% *P. aeruginosa* biofilm growth: 2 µm	BSA Adsorption: 80% *P. aeruginosa* biofilm growth: 5 µm	[133]
UV initiated grafting	AA Ag-MOFs	UF	PWP: 1200 ± 260 Lm^−1^ h^−1^ bar^−1^ Inactivation rate: *E. coli*: 90% *S. aerus*: 95%	PWP: 1500–2500 Lm^−1^ h^−1^ bar^−1^ Inactivation rate: *E. coli*: 0% *S. aerus*: 0%	[129]

**Table 3:** Performance evaluation of modified and pristine PA TFC membranes.

Approach	Modification Materials/Membrane Material	Process	Surface-Grafted Membrane	Pristine Membrane	Ref.
RAFT	pMEDSAH	NF	Na_2_SO_4_ Rejection:70% Flux: 11.5 Lm^−2^ h^−1^ bar^−1^ Fouling resistance, Rr: 35%	Na_2_SO_4_ Rejection: 72% Flux: 1.1 Lm^−2^ h^−1^ bar^−1^ Fouling resistance, Rr: 8%	[138]
ATRP	SPM HEMA	NF	0.01 SPM Rejection: 38.2% Flux: 95 Lm^−2^ h^−1^ 0.01 HEMA Rejection: 31.0% Flux: 105 Lm^−2^ h^−1^	Rejection: 36.1% Flux: 80 Lm^−2^ h^−1^	[137]
ATRP	PAAm	RO	Rejection: 99.2% PWP: 2.4 Lm^−2^ h^−1^ bar^−1^	Rejection: 99.2% PWP: 2.5 Lm^−2^ h^−1^ bar^−1^	[140]
ATRP	PVCIB	RO	Rejection: 98.3% PWP: 5.72 Lm^−2^ h^−1^ bar^−1^ *E. coli* mortality: 98.8%	Rejection: 97.3% PWP: 4.59 Lm^−2^ h^−1^ bar^−1^ *E. coli* mortality: 15.1%	[151]
SI-ATRP	pMEDSAH	RO	Rejection: 94.5% Permeability: 20 Lm^−2^ h^−1^/MPa Bacterial adhesion: 2.0%	Rejection: 96% Permeability: 68 Lm^−2^ h^−1^/MPa Bacterial adhesion: 9.5%	[141]
SI-ATRP	pMEDSAH pTFEMA	RO	Rejection_pMEDSAH_: 95.4% Rejection_pMEDSAH-pTFEMA_: 95.3% Permeability_pMEDSAH_: 3.5 Lm^−2^ h^−1^ bar^−1^ Permeability_pMEDSAH-pTFEMA_: 3.0 Lm^−2^ h^−1^ bar^−1^	Rejection: 96.8% Permeability: 5.3 Lm^−2^ h^−1^ bar^−1^	[142]
SI-ATRP	pHEMA	RO	Rejection: 97% Bacterial adhesion: 2.8%	Rejection: 97% Bacterial adhesion:7.6%	[139]
SI-ATRP	pPEG	RO	Rejection: 97% Bacterial adhesion: 0.5%	Rejection: 97% Bacterial adhesion:7.6%	[139]
SI-ATRP	pMEDSAH	RO	Rejection: 97% Bacterial adhesion: 0.1%	Rejection: 97% Bacterial adhesion:7.6%	[139]
SI-ATRP	CAA TMA	RO	Rejection: 80% Flux: 45 Lm^−2^ h^−1^/MPa	Rejection: 99% Flux: 60 Lm^−2^ h^−1^/MPa	[145]
ATRP	Ag NPs zwitterion	FO	Flux: 1.1 Lm^−2^ h^−1^ bar^−1^ Surface: Smoother EPS Biovolume: 10.7 ± 2.1 µm^3^ µm^−2^	Flux: 1 Lm^−2^ h^−1^ bar^−1^ Surface: Rougher EPS Biovolume: 27.0 ± 3.4 µm^3^ µm^−2^	[152]
ATRP	Silica NPs zwitterion	FO	Surface: Smoother Permeability: 4.8 Lm^−2^ h^−1^ bar^−1^ Attached *E. coli*: 0.1 × 10^5^ cells/cm^2^	Surface: Rougher Permeability: 5.9 Lm^−2^ h^−1^ bar^−1^ Attached *E. coli*: 1.4 × 10^5^ cells/cm^2^	[153]
ATRP	PVI AIBA	FO	Flux: 91.6 Lm^−2^ h^−1^ *E. coli* mortality rate: 98.8%	Flux: 68.7 Lm^−2^ h^−1^ *E. coli* mortality rate: 75.6%	[31]
Plasma initiated grafting	Low pressure NH_3_ plasma	NF	Rejection: 95% Flux: 1.4 Lm^−2^ h^−1^ bar^−1^ BSA Adsorption: 0.22 mg BSA/mg membrane	Rejection: 85% Flux: 1 Lm^−2^ h^−1^ bar^−1^ BSA Adsorption: 0.38 mg BSA/mg membrane	[154]
Plasma initiated grafting	Triglyme	RO	Rejection: 98.1% Flux: 45.5 Lm^−2^ h^−1^	Rejection: 98.5% Flux: 47 Lm^−2^ h^−1^	[155]
Plasma initiated grafting	MA VIM	RO	Rejection: 97% Flux: 49.2 Lm^−2^ h^−1^	Rejection: 98% Flux: 44.9 Lm^−2^ h^−1^	[147]
Plasma initiated grafting	Pure Helium Water	RO	Rejection: 98% Flux: 50 Lm^−2^ h^−1^	Rejection: 98% Flux: 30 Lm^−2^ h^−1^	[147]
Radiation initiated grafting (UV)	AA	NF	Rejection (5 g/L AA,5 min): 43% Flux (5 g/L AA,5 min): 2 Lm^−2^ h^−1^	Rejection: 59% Flux: 0.75 Lm^−2^ h^−1^	[149]
Radiation initiated grafting (UV)	AA	NF	Rejection: 95.8% Na_2_SO_4_ Rejection: 98.2% Flux: 39 Lm^−2^ h^−1^	Rejection: 93% Na_2_SO_4_Rejection: 97.8% Flux: 29 Lm^−2^ h^−1^	[148]
Radiation initiated grafting (UV)	AA	NF	Rejection: 98.5% Flux: 1.05 Lm^−2^ h^−1^ bar^−1^ Irreversible Fouling Factor, FRw: 8% (at pH 7)	Rejection: 98% Flux:1 Lm^−2^ h^−1^ bar^−1^ Irreversible Fouling Factor, FRw: 24% (at pH 7)	[156]
Radiation initiated grafting (UV)	PHMB PEG	NF	Na_2_SO_4_ Rejection: 99.5% FRR:70.8% Bacterial inhibition rate: 98.6%	Na_2_SO_4_ Rejection: 99.5% FRR:44.7% Bacterial inhibition rate:76%	[157]
Radiation initiated grafting (ᵞ-ray)	NVP PVP Cobalt-60	RO	Rejection: 99.5% FRR_BSA_: 91.23%	Rejection: 98.3% FRR_BSA_: 62.28%	[150]
Radiation initiated grafting (ᵞ-ray)	NIPAM Cobalt-60	RO	Rejection: 89% Flux: 8.14 Lm^−2^ h^−1^ bar ^−1^	Rejection: 94% Flux: 9.7 Lm^−2^ h^−1^ bar ^−1^	[158]

## Data Availability

Not applicable.
